# Stem cell-based therapy for human diseases

**DOI:** 10.1038/s41392-022-01134-4

**Published:** 2022-08-06

**Authors:** Duc M. Hoang, Phuong T. Pham, Trung Q. Bach, Anh T. L. Ngo, Quyen T. Nguyen, Trang T. K. Phan, Giang H. Nguyen, Phuong T. T. Le, Van T. Hoang, Nicholas R. Forsyth, Michael Heke, Liem Thanh Nguyen

**Affiliations:** 1 Department of Research and Development, Vinmec Research Institute of Stem Cell and Gene Technology, Vinmec Healthcare System, Hanoi, Vietnam; 2 Department of Cellular Therapy, Vinmec High-Tech Center, Vinmec Healthcare System, Hanoi, Vietnam; 3 Institute for Science & Technology in Medicine, Keele University, Keele, UK; 4 Department of Biology, Stanford University, Stanford, CA USA

**Keywords:** Stem-cell research, Mesenchymal stem cells

## Abstract

Recent advancements in stem cell technology open a new door for patients suffering from diseases and disorders that have yet to be treated. Stem cell-based therapy, including human pluripotent stem cells (hPSCs) and multipotent mesenchymal stem cells (MSCs), has recently emerged as a key player in regenerative medicine. hPSCs are defined as self-renewable cell types conferring the ability to differentiate into various cellular phenotypes of the human body, including three germ layers. MSCs are multipotent progenitor cells possessing self-renewal ability (limited in vitro) and differentiation potential into mesenchymal lineages, according to the International Society for Cell and Gene Therapy (ISCT). This review provides an update on recent clinical applications using either hPSCs or MSCs derived from bone marrow (BM), adipose tissue (AT), or the umbilical cord (UC) for the treatment of human diseases, including neurological disorders, pulmonary dysfunctions, metabolic/endocrine-related diseases, reproductive disorders, skin burns, and cardiovascular conditions. Moreover, we discuss our own clinical trial experiences on targeted therapies using MSCs in a clinical setting, and we propose and discuss the MSC tissue origin concept and how MSC origin may contribute to the role of MSCs in downstream applications, with the ultimate objective of facilitating translational research in regenerative medicine into clinical applications. The mechanisms discussed here support the proposed hypothesis that BM-MSCs are potentially good candidates for brain and spinal cord injury treatment, AT-MSCs are potentially good candidates for reproductive disorder treatment and skin regeneration, and UC-MSCs are potentially good candidates for pulmonary disease and acute respiratory distress syndrome treatment.

## Introduction

The successful approval of cancer immunotherapies in the US and mesenchymal stem cell (MSC)-based therapies in Europe have turned the wheel of regenerative medicine to become prominent treatment modalities.^1–3^ Cell-based therapy, especially stem cells, provides new hope for patients suffering from incurable diseases where treatment approaches focus on management of the disease not treat it. Stem cell-based therapy is an important branch of regenerative medicine with the ultimate goal of enhancing the body repair machinery via stimulation, modulation, and regulation of the endogenous stem cell population and/or replenishing the cell pool toward tissue homeostasis and regeneration.^4^ Since the stem cell definition was introduced with their unique properties of self-renewal and differentiation, they have been subjected to numerous basic research and clinical studies and are defined as potential therapeutic agents. As the main agenda of regenerative medicine is related to tissue regeneration and cellular replacement and to achieve these targets, different types of stem cells have been used, including human pluripotent stem cells (hPSCs), multipotent stem cells and progenitor cells.^5^ However, the emergence of private and unproven clinics that claim the effectiveness of stem cell therapy as “magic cells” has raised highly publicized concerns about the safety of stem cell therapy. The most notable case involved the injection of a cell population derived from fractionated lipoaspirate into the eyes of three patients diagnosed with macular degeneration, resulting in the loss of vision for these patients.^6^ Thus, as regenerative medicine continues to progress and evolve and to clear the myth of the “magic” cells, this review provides a brief overview of stem cell-based therapy for the treatment of human diseases.

**Fig. 1:**
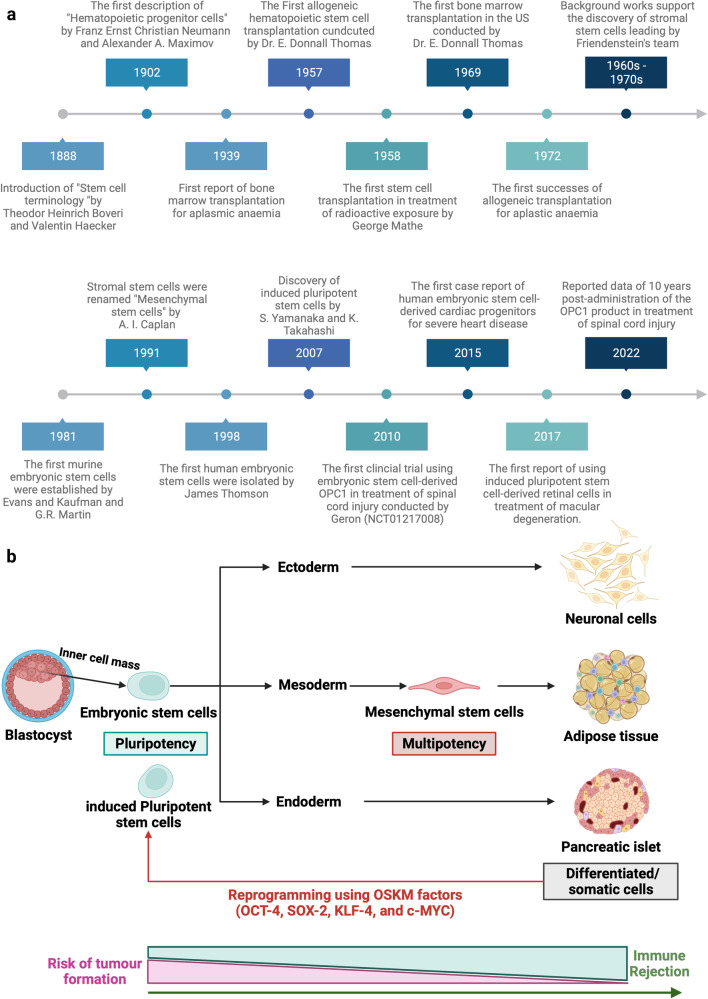
Stem cell-based therapy: the history and cell source. **a** The timeline of major discoveries and advances in basic research and clinical applications of stem cell-based therapy. The term “stem cells” was first described in 1888, setting the first milestone in regenerative medicine. The hematopoietic progenitor cells were first discovered in 1902. In 1939, the first bone marrow transplantation was conducted in the treatment of aplasmic anemia. Since then, the translation of basic research to preclinical studies to clinical trials has driven the development of stem cell-based therapy by many discoveries and milestones. The isolations of “mesenchymal stem cells” in 1991 following by the discovery of human pluripotent stem cells have recently contributed to the progress of stem cell-based therapy in the treatment of human diseases. **b** Schematic of the different cell sources that can be used in stem cell-based therapy. (1) Human pluripotent stem cells, including embryonic stem cells (derived from inner cell mass of blastocyst) and induced pluripotent stem cells confer the ability to proliferate indefinitely in vitro and differentiate into numerous cell types of the human body, including three germ layers. (2) Mesenchymal stem cells are multipotent stem cells derived from mesoderm possessing self-renewal ability (limited in vitro) and differentiation potential into mesenchymal lineages. The differentiated/somatic cells can be reprogrammed back to the pluripotent stage using OSKM factors to generate induced pluripotent stem cells. It is important to note that stem cells show a relatively higher risk of tumor formation and lower risk of immune rejection (in the case of mesenchymal stem cells) when compared to that of somatic cells. The figure was created with BioRender.com

Stem cell therapy is a novel therapeutic approach that utilizes the unique properties of stem cells, including self-renewal and differentiation, to regenerate damaged cells and tissues in the human body or replace these cells with new, healthy and fully functional cells by delivering exogenous cells into a patient.^7^ Stem cells for cell-based therapy can be of (1) autologous, also known as self-to-self therapy, an approach using the patient’s own cells, and (2) allogeneic sources, which use cells from a healthy donor for the treatment.^8^ The term “stem cell” were first used by the eminent German biologist Ernst Haeckel to describe the properties of fertilized egg to give rise to all cells of the organism in 1868.^9^ The history of stem cell therapy started in 1888, when the definition of stem cell was first coined by two German zoologists Theodor Heinrich Boveri and Valentin Haecker,^9^ who set out to identify the distinct cell population in the embryo capable of differentiating to more specialized cells (Fig. 1a). In 1902, studies carried out by the histologist Franz Ernst Christian Neumann, who was working on bone marrow research, and Alexander Alexandrowitsch Maximov demonstrated the presence of common progenitor cells that give rise to mature blood cells, a process also known as haematopoiesis.^10^ From this study, Maximov proposed the concept of polyblasts, which later were named stem cells based on their proliferation and differentiation by Ernst Haeckel.^11^ Maximov described a hematopoietic population presented in the bone marrow. In 1939, the first case report described the transplantation of human bone marrow for a patient diagnosed with aplastic anemia. Twenty years later, in 1958, the first stem cell transplantation was performed by the French oncologist George Mathe to treat six nuclear researchers who were accidentally exposed to radioactive substances using bone marrow transplantation.^12^ Another study by George Mathe in 1963 shed light on the scientific community, as he successfully conducted bone marrow transplantation in a patient with leukemia. The first allogeneic hematopoietic stem cell transplantation (HSCT) was pioneered by Dr. E. Donnall Thomas in 1957.^13^ In this initial study, all six patients died, and only two patients showed evidence of transient engraftment due to the unknown quantities and potential hazards of bone marrow transplantation at that time. In 1969, Dr. E. Donnall Thomas conducted the first bone marrow transplantation in the US, although the success of the allogeneic treatment remained exclusive. In 1972, the year marked the discovery of cyclosporine (the immune suppressive drug),^14^ the first successes of allogeneic transplantation for aplastic anemia and acute myeloid leukemia were reported in a 16-year-old girl.^15^ From the 1960s to the 1970s, series of works conducted by Friendenstein and coworkers on bone marrow aspirates demonstrated the relationship between osteogenic differentiation and a minor subpopulation of cells derived from bone marrow.^16^ These cells were later proven to be distinguishable from the hematopoietic population and to be able to proliferate rapidly as adherent cells in tissue culture vessels. Another important breakthrough from Friendenstein’s team was the discovery that these cells could form the colony-forming unit when bone marrow was seeded as suspension culture following by differentiation into osteoblasts, adipocytes, and chondrocytes, suggesting that these cells confer the ability to proliferate and differentiate into different cell types.^17^ In 1991, combined with the discovery of human embryonic stem cells (hESCs), which will be discussed in the next section, the term “mesenchymal stem cells”, previously known as stromal stem cells or “osteogenic” stem cells, was first coined in Caplan and widely used to date.^18^ Starting with bone marrow transplantation 60 years ago, the journey of stem cell therapy has developed throughout the years to become a novel therapeutic agent of regenerative medicine to treat numerous incurable diseases, which will be reviewed and discussed in this review, including neurological disorders, pulmonary dysfunctions, metabolic/endocrine-related diseases, reproductive disorders, skin burns, and cardiovascular conditions).

In this review, we described the different types of stem cell-based therapies (Fig. 1b), including hPSCs and MSCs, and provided an overview of their definition, history, and outstanding clinical applications. In addition, we further created the first literature portfolio for the “targeted therapy” of MSCs based on their origin, delineating their different tissue origins and downstream applications with an in-depth discussion of their mechanism of action. Finally, we provide our perspective on why the tissue origin of MSCs could contribute greatly to their downstream applications as a proposed hypothesis that needs to be proven or disproven in the future to further enhance the safety and effectiveness of stem cell-based therapy.

## Stem cell-based therapy: an overview of current clinical applications

### Cardiovascular diseases

The clinical applications of stem cell-based therapies for heart diseases have been recently discussed comprehensively in the reviews^19,20^ and therefore will be elaborated in this study as the focus discussions related to hPSCs and MSCs in the following sections. In general, the safety profiles of stem cell-based therapies are supported by a large body of preclinical and clinical studies, especially adult stem cell therapy (such as MSC-based products). However, clinical trials have not yet yielded data supporting the efficacy of the treatment, as numerous studies have shown paradoxical results and no statistically significant differences in infarct size, cardiac function, or clinical outcomes, even in phase III trials.^21^ The results of a meta-analysis showed that stem cells derived from different sources did not exhibit any therapeutic effects on the improvement of myocardial contractility, cardiovascular remodeling, or clinical outcomes.^22^ The disappointing results obtained from the clinical trials thus far could be explained by the fact that the administered cells may exert their therapeutic effects via an immune modulation rather than regenerative function. Thus, well-designed, randomized and placebo-controlled phase III trials with appropriate cell-preparation methods, patient selection, follow-up schedules and suitable clinical measurements need to be conducted to determine the efficacy of the treatments. In addition, concerns related to optimum cell source and dose, delivery route and timing of administration, cell distribution post administration and the mechanism of action also need to be addressed. In the following section of this review, we present clinical trials related to MSC-based therapy in cardiovascular disease with the aim of discussing the contradictory results of these trials and analyzing the potential challenges underlying the current approaches.

### Digestive system diseases

Gastrointestinal diseases are among the most diagnosed conditions in the developed world, altering the life of one-third of individuals in Western countries. The gastrointestinal tract is protected from adverse substances in the gut environment by a single layer of epithelial cells that are known to have great regenerative ability in response to injuries and normal cell turnover.^23^ These epithelial cells have a rapid turnover rate of every 2–7 days under normal conditions and even more rapidly following tissue damage and inflammation. This rapid proliferation ability is possible owing to the presence of a specific stem cell population that is strictly compartmentalized in the intestinal crypts.^24^ The gastrointestinal tract is highly vulnerable to damage, tissue inflammation and diseases once the degradation of the mucosal lining layer occurs. The exposure of intestinal stem cells to the surrounding environment of the gut might result in the direct destruction of the stem cell layer or disruption of intestinal functions and lead to overt clinical symptoms.^25^ In addition, the accumulation of stem cell defects as well as the presence of chronic inflammation and stress also contributes to the reduction of intestinal stem cell quality.

In terms of digestive disorders, Crohn’s disease (CD) and ulcerative colitis are the two major forms of inflammatory bowel disease (IBD) and represent a significant burden on the healthcare system. The former is a chronic, uncontrolled inflammatory condition of the intestinal mucosa characterized by segmental transmural mucosal inflammation and granulomatous changes.^26^ The latter is a chronic inflammatory bowel disease affecting the colon and rectum, characterized by mucosal inflammation initiating in the rectum and extending proximal to the colon in a continuous fashion.^27^ Cellular therapy in the treatment of CD can be divided into haematopoietic stem cell-based therapy and MSC-based therapy. The indication and recommendation of using HSCs for the treatment of IBD were proposed in 1995 by an international committee with four important criteria: (1) refractory to immunosuppressive treatment; (2) persistence of the disease conditions indicated via endoscopy, colonoscopy or magnetic resonance enterography; (3) patients who underwent an imminent surgical procedure with a high risk of short bowel syndromes or refractory colonic disease; and (4) patients who refused to treat persistent perianal lesions using coloproctectomy with a definitive stroma implant.^28^ In the standard operation procedure, patents’ HSCs were recruited using cyclophosphamide, which is associated with granulocyte colony-stimulating factor (G-CSF), at two different administered concentrations (4 g/m^2^ and 2 g/m^2^). Recently, it was reported that high doses of cyclophosphamide do not improve the number of recruited HSCs but increase the risk of cardiac and bladder toxicity. An interest in using HSCTs in CD originated from case reports that autologous HSCTs can induce sustained disease remission in some^29,30^ but not all patients^31–33^ with CD. The first phase I trial was conducted in Chicago and recruited 12 patients with active moderate to severe CD refractory to conventional therapies. Eleven of 12 patients demonstrated sustained remission after a median follow-up of 18.5 months, and one patient developed recurrence of active CD.^31^ The ASTIC trial (the Autologous Stem Cell Transplantation International Crohn Disease) was the first randomized clinical trial with the largest cohort of patients undergoing HSCT for refractory CD in 2015.^34^ The early report of the trial showed no statistically significant improvement in clinical outcomes of mobilization and autologous HSCT compared with mobilization followed by conventional therapy. In addition, the procedure was associated with significant toxicity, leading to the suggestion that HSCT for patients with refractory CD should not be widespread. Interestingly, by using conventional assessments for clinical trials for CD, a group reassessed the outcomes of patients enrolled in the ASTIC trial showing clinical and endoscopic benefits, although a high number of adverse events were also detected.^35^ A recent systematic review evaluated 18 human studies including 360 patients diagnosed with CD and showed that eleven studies confirmed the improvement of Crohn’s disease activity index between HSCT groups compared to the control group.^36^ Towards the cell sources, HSCs are the better sources as they afforded more stable outcomes when compared to that of MSC-based therapy.^37^ Moreover, autologous stem cells were better than their allogeneic counterparts.^36^ The safety of stem cell-based therapy in the treatment of CD has attracted our attention, as the risk of infection in patients with CD was relatively higher than that in those undergoing administration to treat cancer or other diseases. During the stem cell mobilization process, patient immunity is significantly compromised, leading to a high risk of infection, and requires carefully nursed and suitable antibiotic treatment to reduce the development of adverse events. Taken together, stem cell-based therapy for digestive disease reduced inflammation and improved the patient’s quality of life as well as bowel functions, although the high risk of adverse events needs to be carefully monitored to further improve patient safety and treatment outcomes.

### Liver diseases

The liver is the largest vital organ in the human body and performs essential biological functions, including detoxification of the organism, metabolism, supporting digestion, vitamin storage, and other functions.^38^ The disruption of liver homeostasis and function might lead to the development of pathological conditions such as liver failure, cirrhosis, cancer, alcoholic liver disease, nonalcoholic fatty liver disease (NAFLD), and autoimmune liver disease (ALD). Orthotropic liver transplantation is the only effective treatment for severe liver diseases, but the number of available and suitable donor organs is very limited. Currently, stem cell-based therapies in the treatment of liver disease are associated with HSCs, MSCs, hPSCs, and liver progenitor cells.

Liver failure is a critical condition characterized by severe liver dysfunctions or decompensation caused by numerous factors with a relatively high mortality rate. Stem cell-based therapy is a novel alternative approach in the treatment of liver failure, as it is believed to participate in the enhancement of liver regeneration and recovery. The results of a meta-analysis including four randomized controlled trials and six nonrandomized controlled trials in the treatment of acute-on-chronic liver failure (ACLF) demonstrated that clinical outcomes of stem cell therapy were achieved in the short term, requiring multiple doses of stem cells to prolong the therapeutic effects.^39,40^ Interestingly, although MSC-based therapies improved liver functions, including the model of end-stage liver disease score, albumin level, total bilirubin, and coagulation, beneficial effects on survival rate and aminotransferase level were not observed.^41^ A randomized controlled trial illustrated the improvement of liver functions and reduction of severe infections in patients with hepatitis B virus-related ACLF receiving allogeneic bone marrow-derived MSCs (BM-MSCs) via peripheral infusion.^42^ HSCs from peripheral blood after the G-CSF mobilization process were used in a phase I clinical trial and exhibited an improvement in serum bilirubin and albumin in patients with chronic liver failure without any specific adverse events related to the administration.^43^ Taken together, an overview of stem cell-based therapy in the treatment of liver failure indicates the potential therapeutic effects on liver functions with a strong safety profile, although larger randomized controlled trials are still needed to assure the conclusions.

Liver cirrhosis is one of the major causes of morbidity and mortality worldwide and is characterized by diffuse nodular regeneration with dense fibrotic septa and subsequent parenchymal extinction leading to the collapse of liver vascular structure.^44^ In fact, liver cirrhosis is considered the end-stage of liver disease that eventually leads to death unless liver transplantation is performed. Stem cell-based therapy, especially MSCs, currently emerges as a potential treatment with encouraging results for treating liver cirrhosis. In a clinical trial using umbilical cord-derived MSCs (UC-MSCs), 45 chronic hepatitis B patients with decompensated liver cirrhosis were divided into two groups: the MSC group (*n* = 30) and the control group (*n* = 15).^45^ The results showed a significant reduction in ascites volume in the MSC group compared with the control. Liver function was also significantly improved in the MSC groups, as indicated by the increase in serum albumin concentration, reduction in total serum bilirubin levels, and decrease in the sodium model for end-stage liver disease score.^45^ Similar results were also reported from a phase II trial using BM-MSCs in 25 patients with HCV-induced liver cirrhosis.^46^ Consistent with these studies, three other clinical trials targeting liver cirrhosis caused by hepatitis B and alcoholic cirrhosis were conducted and confirmed that MSC administration enhanced and recovered liver functions.^47–49^ With the large cohort study as the clinical trial conducted by Fang, the safety and potential therapeutic effects of MSC-based therapies could be further strengthened and confirmed the feasibility of the treatment in virus-related liver cirrhosis.^49^ In terms of delivery route, a randomized controlled phase 2 trial suggested that systemic delivery of BM-MSCs does not show therapeutic effects on patients with liver cirrhosis.^50^ MSCs are not the only cell source for liver cirrhosis. Recently, an open-label clinical trial conducted in 19 children with liver cirrhosis due to biliary atresia after the Kasai operation illustrated the safety and feasibility of the approach by showing the improvement of liver function after bone marrow mononuclear cell (BMNC) administration assessed by biochemical tests and pediatric end-stage liver disease (PELD) scores.^51^ Another study using BMNCs in 32 decompensated liver cirrhosis patients illustrated the safety and effectiveness of BMNC administration in comparison with the control group.^52^ Recently, a long-term analysis of patients receiving peripheral blood-derived stem cells indicated a significant improvement in the long-term survival rate when compared to the control group, and the risk of hepatocellular carcinoma formation did not increase.^53^ CD133^+^ HSC infusion was performed in a multicentre, open, randomized controlled phase 2 trial in patients with liver cirrhosis; the results did not support the improvement of liver conditions, and cirrhosis persisted.^54^ Notably, these results are in line with a previous randomized controlled study, which also reported that G-CSF and bone marrow-derived stem cells delivered via the hepatic artery did not introduce therapeutic potential as expected.^55^ Thus, stem cell-based therapy for liver cirrhosis is still in its immature stage and requires larger trials with well-designed experiments to confirm the efficacy of the treatment.

Nonalcoholic fatty liver disease (NAFLD) is the most common medical condition caused by genetic and lifestyle factors and results in a severe liver condition and increased cardiovascular risk.^56^ NAFLD is the hidden enemy, as most patients are asymptomatic for a long time, and their routine life is unaffected. Thus, the detection, identification, and management of NAFLD conditions are challenging tasks, as patients diagnosed with NAFLD often develop nonalcoholic steatohepatitis, cirrhosis, and hepatocellular carcinoma.^57^ Although preclinical studies have shown that stem cell administration could enhance liver function in NAFLD models, a limited number of clinical trials were performed in human subjects. Recently, a multi-institutional clinical trial using freshly isolated autologous adipose tissue-derived regenerative cells was performed in Japan to treat seven NAFLD patients.^58^ The results illustrated the improvement in the serum albumin level of six patients and prothrombin activity of five patients, and no treatment-related adverse events or severe adverse events were observed. This study illustrates the therapeutic potential of stem cell-based therapy in the treatment of NAFLD.

Autoimmune liver disease (ALD) is a severe liver condition affecting children and adults worldwide, with a female predominance.^59^ The condition occurs in genetically predisposed patients when a stimulator, such as virus infection, leads to a T-cell-mediated autoimmune response directed against liver autoantigens. As a result, patients with ALD might develop liver cirrhosis, hepatocellular carcinoma, and, in severe cases, death. To date, HSCT and bone marrow transplantation are the two common stem cell-based therapies exhibiting therapeutic potential for ALD in clinical trials. An interesting report illustrated that haploidentical HSCTs could cure ALD in patients with sickle cells.^60^ This report is particularly important, as it illustrates the potential therapeutic approach of using haploidentical HSCTs to treat patients with both sickle cells and ALD. Another case report described a 19-year-old man with a 4-year history of ALD who developed acute lymphoblastic leukemia and required allogeneic bone marrow transplantation from this wholesome brother.^61^ The clinical data showed that immunosuppressive therapy for transplantation generated ALD remission in the patient.^62^ However, the data also provided valid information related to the sustained remission and the normalization of ASGPR-specific suppressor-inducer T-cell activity following bone marrow transplantation, suggesting that these suppressor functions originated from donor T cells.^61^ Thus, it was suggested that if standard immunosuppressive treatment fails, alternative cellular immunotherapy would be a viable option for patients with ALD. Primary biliary cholangitis (PBC), usually known as primary biliary cirrhosis, is a type of ALD characterized by a slow, progressive destruction of small bile ducts of the liver leading to the formation of cirrhosis and accumulation of bile and other toxins in the liver. A pilot, single-arm trial from China recruited seven patents with PBC who had a suboptimal response to ursodeoxycholic acid (UDCA) treatment.^63^ These patients received UDCA treatment in combination with three rounds of allogeneic UC-MSCs at 4-week intervals with a dose of 0.5 × 10^6^ cells/kg of patient body weight via the peripheral vein. No treatment-related adverse events or severe adverse events were observed throughout the course of the study. The clinical data indicated significant improvement in liver function, including reduction of serum ALP and gamma-glutamyltransferase (GGT) at 48 weeks post administration. The common symptoms of PBC, including fatigue, pruritus, and hypogastric ascites volume, were also reduced, supporting the feasibility of MSC-based therapy in the treatment of PBC, although major limitations of the study were nonrandomized, no control group and small sample size. Another study was conducted in China with ten PBC patients who underwent incompetent UDCA treatment for more than 1 year. These patients received a range of 3–5 allogeneic BM-MSCs/kg body weight by intravenous infusion.^64^ Although these early studies have several limitations, such as small sample size, nonrandomization, and no control group, their preliminary data related to safety and efficacy herald the prospects and support the feasibility of stem cell-based therapy in the treatment of ALD.

In summary, the current number of trials for liver disease using stem cell-based therapy has provided fundamental data supporting the safety and potential therapeutic effects in various liver diseases. Unfortunately, due to the small number of trials, several obstacles need to be overcome to prove the effectiveness of the treatments, including (1) stem cell source and dose, (2) administration route, (3) time of intervention, and (4) clinical assessments during the follow-up period. Only by addressing these challenges we will be able to prove, facilitate and promote stem cell-based therapy as a mainstream treatment for liver diseases.

### Arthritis

Arthritis is a general term describing cartilage conditions that cause pain and inflammation of the joints. Osteoarthritis (OA) is the most common form of arthritis caused by persistent degeneration and poor recovery of articular cartilage.^65^ OA affects one or several diarthrodial joints, such as small joints at the hand and large joints at the knee and hips, leading to severe pain and subsequent reduction in the mobility of patients. There are two types of OA: (1) primary OA or idiopathic OA and secondary OA caused by causative factors such as trauma, surgery, and abnormal joint development at birth.^66^ As conventional treatments for OA are not consistent in their effectiveness and might cause unbearable pain as well as long-term rehabilitation (in the case of joint replacement), there is a need for a more reliable, less painful, and curative therapy targeting the root of OA.^67^ Thus, stem cell therapy has recently emerged as an alternative approach for OA and has drawn great attention in the regenerative field.

The administration of HSCs has been proven to reduce bone lesions, enhance bone regeneration and stimulate the vascularization process in degenerative cartilage. Attempts were made to evaluate the efficacy of peripheral blood stem cells in ten OA patients by three intraarticular injections. Post-administration analysis indicated a reduction in the WOMAC index with a significant reduction in all parameters. All patients completed 6-min walk tests with an increase of more than 54 meters. MRI analysis indicated an improvement in cartilage thickness, suggesting that cartilage degeneration was reduced post administration. To further enhance the therapeutic potential of HSCT, CD34^+^ stem cells were proposed to be used in combination with the rehabilitation algorithm, which included three stages: preoperative, hospitalization and outpatient periods.^68^ Currently, a large wave of studies has been directed to MSC-based therapy for the treatment of OA due to their immunoregulatory functions and anti-inflammatory characteristics. MSCs have been used as the main cell source in several multiple and small-scale trials, proving their safety profile and potential effectiveness in alleviating pain, reducing cartilage degeneration, and enhancing the regeneration of cartilage structure and morphology in some cases. However, the best source of MSCs, whether from bone marrow, adipose tissue, or umbilical cord, for the management of OA is still a great question to be answered. A systematic review investigating over sixty-one of 3172 articles with approximately 2390 OA patients supported the positive effects of MSC-based therapy on OA patients, although the study also pointed out the fact that these therapeutic potentials were based on limited high-quality evidence and long-term follow-up.^69^ Moreover, the study found no obvious evidence supporting the most effective source of MSCs for treating OA. Another systematic review covering 36 clinical trials, of which 14 studies were randomized trials, provides an interesting view in terms of the efficacy of autologous MSC-based therapy in the treatment of OA.^70^ In terms of BM-MSCs, 14 clinical trials reported the clinical outcomes at the 1-year follow-up, in which 57% of trials reported clinical outcomes that were significantly better in comparison with the control group. However, strength analysis of the data set showed that outcomes from six trials were low, whereas the outcomes of the remaining eight trials were extremely low. Moreover, the positive evidence obtained from MRI analysis was low to very low strength of evidence after 1-year post administration.^70^ Similar results were also found in the outcome analysis of autologous adipose tissue-derived MSCs (AT-MSCs). Thus, the review indicated low quality of evidence for the therapeutic potential of MSC therapy on clinical outcomes and MRI analysis. The low quality of clinical outcomes could be explained by the differences in interventions (including cell sources, cell doses, and administration routes), combination treatments (with hyaluronic acid,^71^ peripheral blood plasma,^72^ etc.), control treatments and clinical outcome measurements between randomized clinical trials.^73^ In addition, the data of the systematic analysis could not prove the better source of MSCs for OA treatment. Taken together, although stem cell-based therapy has been shown to be safe and feasible in the management of OA, the authors support the notion that stem cell-based therapy could be considered an alternative treatment for OA when first-line treatments, such as education, exercise, and body weight management, have failed.

### Cancer treatment

Stem cell therapy in the treatment of cancer is a sensitive term and needs to be used and discussed with caution. Clinicians and researchers should protect patients with cancer from expensive and potentially dangerous or ineffective stem cell-based therapy and patients without a cancer diagnosis from the risk of malignancy development. In general, unproven stem cell clinics employed three cell-based therapies for cancer management, including autologous HSCTs, stromal vascular fraction (SVF), and multipotent stem cells, such as MSCs. Allogeneic HSCTs confer the ability to generate donor lymphocytes that contribute to the suppression and regression of hematological malignancies and select solid tumors, a specific condition known as “graft-versus-tumor effects”.^74^ However, stem cell clinics provide allogeneic cell-based therapy for the treatment of solid malignancies despite limited scientific evidence supporting the safety and efficacy of the treatment. High-quality evidence from the Cochrane library shows that marrow transplantation via autologous HSCTs in combination with high-dose chemotherapy does not improve the overall survival of women with metastatic breast cancer. In addition, a study including more than 41,000 breast cancer patients demonstrated no significant difference in survival benefits between patients who received HSCTs following high-dose chemotherapy and patients who underwent conventional treatment.^75^ Thus, the use of autologous T-cell transplants as monotherapy and advertising stem cell-based therapies as if they are medically approved or preferred treatment of solid tumors is considered untrue statements and needs to be alerted to cancer patients.^76^

Over the past decades, many preclinical studies have demonstrated the potential of MSC-based therapy in cancer treatment due to their unique properties. They confer the ability to migrate toward damaged sites via inherent tropism controlled by growth factors, chemokines, and cytokines. MSCs express specific C–X–C chemokine receptor type 4 (CXCR4) and other chemokine receptors (including CCR1, CCR2, CCR4, CCR7, etc.) that are essential to respond to the surrounding signals.^77^ In addition, specific adherent proteins, including CD49d, CD44, CD54, CD102, and CD106, are also expressed on the MSC surface, allowing them to attach, rotate, migrate, and penetrate the blood vessel lumen to infiltrate the damaged tissue.^78^ Similar to damaged tissues, tumors secrete a wide range of chemoattractant that also attract MSC migration via the CXCL12/CXCR4 axis. Previous studies also found that MSC migration toward the cancer site is tightly controlled by diffusible cytokines such as interleukin 8 (IL-8) and growth factors including transforming growth factor-beta 1 (TGF-β1),^79^ platelet-derived growth factor (PDGF),^80^ fibroblast growth factor 2 (FGF-2),^81^ vascular endothelial growth factor (VEGF),^81^ and extracellular matrix molecules such as matrix metalloproteinase-2 (MMP-2).^82^ Once MSCs migrate successfully to cancerous tissue, accumulating evidence demonstrates the interaction between MSCs and cancer cells to exhibit their protumour and antitumour effects, which are the major concerns of MSC-based therapy. MSCs are well-known for their regenerative effects that regulate tissue repair and recovery. This unique ability is also attributed to the protumour functions of these cells. A previous study reported that breast cancer cells induce MSC secretion of chemokine (C–C motif) ligand 5 (CCL-5), which regulates the tumor invasion process.^83,84^ Other studies also found that MSCs secrete a wide range of growth factors (VEGF, basic FGF, HGF, PDGF, etc.) that inhibits apoptosis of cancer cells.^85^ Moreover, MSCs also respond to signals released from cancer cells, such as TGF-β,^86^ to transform into cancer-associated fibroblasts, a specific cell type residing within the tumor microenvironment capable of promoting tumorigenesis.^87^ Although MSCs have been proven to be involved in protumour activities, they also have potent tumor suppression abilities that have been used to develop cancer treatments. It has been suggested that MSCs exhibit their tumor inhibitory effects by inhibiting the Wnt and AKT signaling pathways,^88^ reducing the angiogenesis process,^89^ stimulating inflammatory cell infiltration,^90^ and inducing tumor cell cycle arrest and apoptosis.^91^ To date, the exact functions of MSCs in both protumour and antitumor activities are still a controversial issue across the stem cell field. Other approaches exploit gene editing and tissue engineering to convert MSCs into “a Trojan horse” that could exhibit antitumor functions. In addition, MSCs can also be modified to express specific anticancer miRNAs exhibiting tumor-suppressive behaviors.^92^ However, genetically modified MSCs are still underdeveloped and require intensive investigation in the clinical setting.

To date, ~25 clinical trials have been registered on ClinicalTrials.gov aimed at using MSCs as a therapeutic treatment for cancer.^93^ These trials are mostly phase 1 and 2 studies focusing on evaluating the safety and efficacy of the treatment. Studies exploiting MSC-based therapy have combined MSCs with an oncolytic virus approach. Oncolytic viruses are specific types of viruses that can be genetically engineered or naturally present, conferring the ability to selectively infect cancer cells and kill them without damaging the surrounding healthy cells.^94^ A completed phase I/II study using BM-MSCs infected with the oncolytic adenovirus ICOVIR5 in the treatment of metastatic and refractory solid tumors in children and adult patients demonstrated the safety of the treatment and provided preliminary data supporting their therapeutic potential.^95^ The same group also reported a complete disappearance of all signs of cancer in response to MSC-based therapy in one pediatric case three years post administration.^96^ A reported study in 2019 claimed that adipose-derived MSCs infected with vaccinia virus have the potential to eradicate resistant tumor cells via the combination of potent virus amplification and senitization of the tumor cells to virus infection.^97^ However, in a recently published review, a valid question was posed regarding the 2019 study that “do these reported data merit inclusion in the publication record when they were collected by such groups using a dubious therapeutic that was eventually confiscated by US Marshals?”^76^

Taken together, cancer research and therapy have entered an innovative and fascinating era with advancements in traditional therapies such as chemotherapy, radiotherapy, and surgery on one hand and stem cell-based therapy on the other hand. Although stem cell-based therapy has been considered a novel and attractive therapeutic approach for cancer treatment, it has been hampered by contradictory results describing the protumour and antitumour effects in preclinical studies. Despite this contradictory reality, the use of stem cell-based therapy, especially MSCs, offers new hope to cancer patients by providing a new and more effective tool in personalized medicine. The authors support the use of MSC-based therapy as a Trojan horse to deliver specific anticancer functions toward cancer cells to suppress their proliferation, eradicate cancer cells, or limit the vascularization process of cancerous tissue to improve the clinical safety and efficacy of the treatment.

## Human pluripotent stem cell-based therapy: a growing giant

The discovery of hPSCs, including human embryonic stem cells (hESCs) and human induced pluripotent stem cells (hiPSCs), has revolutionized stem cell research and cell-based therapy.^98^ hESCs were first isolated from blastocyst-stage embryos in 1998,^99^ followed by breakthrough reprogramming research that converted somatic cells into hiPSCs using just four genetic factors.^100,101^ Methods have been developed to maintain these cells long-term in vitro and initiate their differentiation into a wide variety of cell types, opening a new era in regenerative medicine, particularly cell therapy to replace lost or damaged tissues.

### History of hPSCs

hPSCs are defined as self-renewable cell types that confer the ability to differentiate into various cellular phenotypes of the human body, including three germ layers.^102^ Historically, the first pluripotent cell lines to be generated were embryonic carcinoma (EC) cell lines established from human germ cell tumors^103^ and murine undifferentiated compartments.^104^ Although EC cells are a powerful tool in vitro, these cells are not suitable for clinical applications due to their cancer-derived origin and aneuploidy genotype.^105^ The first murine ESCs were established in 1981 based on the culture techniques obtained from EC research.^106^ Murine ESCs are derived from the inner cell mass (ICM) of the pre-implantation blastocyst, a unique biological structure that contains outer trophoblast layers that give rise to the placenta and ICM.^107^ In vivo ESCs only exist for a short period during the embryo’s development, and they can be isolated and maintained indefinitely in vitro in an undifferentiated state. The discovery of murine ESCs has dramatically changed the field of biomedical research and regenerative medicine over the last 40 years. Since then, enormous investigations have been made to isolate and culture ESCs from other species, including hESCs, in 1998.^99^ The success of Thomson et al. in 1998 triggered the great controversy in media and ethical research boards across the globe, with particularly strong objections being raised to the use of human embryos for research purposes.^108^ Several studies using hESCs have been conducted demonstrating their therapeutic potential in the clinical setting. However, the use of hESCs is limited due to (1) the ethical barrier related to the destruction of human embryos and (2) the potential risk of immunological rejection, as hESCs are isolated from pre-implantation blastocysts, which are not autologous in origin. To overcome these two great obstacles, several research groups have been trying to develop technology to generate hESCs, including nuclear transfer technology, the well-known strategy that creates Dolly sheep, although the generation of human nuclear transfer ESCs remains technically challenging.^109^ Taking a different approach, in 2006, Yamanaka and Takahashi generated artificial PSCs from adult and embryonic mouse somatic cells using four transcription factors (*Oct-3/4*, *Sox2*, *Klf4*, and *c-Myc*, called OSKM) reduced from 24 factors.^100^ Thereafter, in 2007, Takahashi and colleagues successfully generated the first hiPSCs exhibiting molecular and biological features similar to those of hESCs using the same OSKM factors.^101^ Since then, hiPSCs have been widely studied to expand our knowledge of the pathogenesis of numerous diseases and aid in developing new cell-based therapies as well as personalized medicine.

### Clinical applications of hPSCs

Since its beginning 24 years ago, hPSC research has evolved momentously toward applications in regenerative medicine, disease modeling, drug screening and discovery, and stem cell-based therapy. In clinical trial settings, the uses of hESCs are restricted by ethical concerns and tight regulation, and the limited preclinical data support their therapeutic potential. However, it is important to acknowledge several successful outcomes of hESC-based therapies in treating human diseases. In 2012, Steven Schwartz and his team reported the first clinical evidence of using hESC-derived retinal pigment epithelium (RPE) in the treatment of Stargardt’s macular dystrophy, the most common pediatric macular degeneration, and an individual with dry age-related macular degeneration.^110,111^ With a differentiation efficiency of RPE greater than 99%, 5 × 10^4^ RPEs were injected into the subretinal space of one eye in each patient. As the hESC source of RPE differentiation was exposed to mouse embryonic stem cells, it was considered a xenotransplantation product and required a lower dose of immunosuppression treatment. This study showed that hESCs improved the vision of patients by differentiating into functional RPE without any severe adverse events. The trial was then expanded into two open-label, phase I/II studies with the published results in 2015 supporting the primary findings.^112^ In these trials, patients were divided into three groups receiving three different doses of hESC-derived RPE, including 10 × 10^4^, 15 × 10^4^ and 50 × 10^4^ RPE cells per eye. After 22 months of follow-up, 19 patients showed improvement in eyesight, seven patients exhibited no improvement, and one patient experienced a further loss of eyesight. The technical challenge of hESC-derived RPE engraftment was an unbalanced proliferation of RPE post administration, which was observed in 72% of treated patients. A similar approach was also conducted in two South Korean patients diagnosed with age-induced macular degeneration and two patients with Stargardt macular dystrophy.^113^ The results supported the safety of hESC-derived RPE cells and illustrated an improvement in visual acuity in three patients. Recently, clinical-graded hESC-derived RPE cells were also developed by Chinese researchers under xeno-free culture conditions to treat patients with wet age-related degeneration.^114^ As hESC development is still associated with ethical concerns and immunological complications related to allogeneic administration, hiPSC-derived RPE cells have emerged as a potential cell source for macular degeneration. Although RPE differentiation protocols have been developed and optimized to improve the efficacy of hiPSC-derived RPE cells, they are still insufficient, time-consuming and labor intensive.^115,116^ For clinical application, an efficient differentiation of “primed” to “naïve” state hiPSCs toward the RPE was developed using feeder-free culture conditions utilizing the transient inhibition of the FGF/MAPK signaling pathway.^117^ Overexpression of specific transcription factors in hiPSCs throughout the differentiation process is also an interesting approach to generate a large number of RPE cells for clinical use. In a recent study, overexpression of three eye-field transcription factors, including *OTX2, PAX6,* and *MITF*, stimulated RPE differentiation in hiPSCs and generated functional RPE cells suitable for transplantation.^118^ To date, although reported data from phase I/II clinical trials have been produced enough to support the safety of hESC-derived RPE cells, the treatment is still in its immature stage. Thus, future studies should focus on the development of the cellular manufacturing process of RPE and the subretinal administration route to further improve the outcomes of RPE fabrication and engraftment into the patient’s retina (recommended review^119^).

Numerous studies have demonstrated that hESC-derived cardiomyocytes exhibit cardiac transcription factors and display a cardiomyocyte phenotype and immature electrical phenotype. In addition, using hPSC-derived cardiomyocytes could provide a large number of cells required for true remuscularization and transplantation. Thus, these cells can be a promising novel therapeutic approach for the treatment of human cardiovascular diseases. In a case report, hESC-derived cardiomyocytes showed potential therapeutic effects in patients with severe heart failure without any subsequent complications.^120^ This study was a phase I trial (ESCORT [Transplantation of Human Embryonic Stem Cell-derived Progenitors in Severe Heart Failure] trial) to evaluate the safety of cardiomyocyte progenitor cells derived from hESCs seeded in fibrin gel scaffolds for 10 patients with severe heart failure (NCT02057900). The encouraging results from this study demonstrated the feasibility of producing hESC-derived cardiomyocyte progenitor cells toward clinical-grade standards and combining them with a tissue-engineered scaffold to treat severe heart disease (the first patient of this trial has already reached the 7-year follow-up in October 2021).^121^ Currently, the two ongoing clinical trials using hPSC-derived cardiomyocytes have drawn great attention, as their results would pave the way to lift the bar for approving therapies for commercial use. The first trial was conducted by a team led by cardiac surgeon Yoshiki Sawa at Osaka University using hiPSC-derived cardiomyocytes embedded in a cell sheet for engraftment (jRCT2052190081). The trials started first with three patients followed by ten patients to assess the safety of the approach. Once safety is met, the treatment can be sold commercially under Japan’s fast-track system for regenerative medicine.^122^ Another trial used a collagen-based construct called BioVAT-HF to contain hiPSC-derived cardiomyocytes. The trial was divided into two parts to evaluate the cell dose: (Part A) recruiting 18 patients and (Part B) recruiting 35 patients to test a broad range of engineered human myocardium (EHM) doses. The expected results from this study will provide the “proof-of-concept” for the use of EHM in the stimulation of heart remuscularization in humans. To date, no adverse events or severe adverse events have been reported from these trials, supporting the safety of the procedure. However, as the number of treated patients was relatively small, limitations in drawing conclusions regarding efficacy are not yet possible.^21,123^

One of the first clinical trials using hPSC-based therapy was conducted by Geron Corporation in 2010 using hESC-derived oligodendrocyte progenitor cells (OPC1) to treat spinal cord injury (SCI). The results confirmed the safety one year post administration in five participants, and magnetic resonance imaging demonstrated improvement of spinal cord deterioration in four participants.^124^ Asterias Biotherapeutic (AST) continued the Geron study by conducting the SCiStar Phase I/IIa study to evaluate the therapeutic effects of AST-OPC1 (NCT02302157). The trial’s results published in clinicaltrials.gov demonstrated significant improvement in running speed, forelimb stride length, forelimb longitudinal deviations, and rear stride frequency. Interestingly, the recently published data of a phase 1, multicentre, nonrandomized, single-group assignment, interventional trial illustrated no evidence of neurological decline, enlarging masses, further spinal cord damage, or syrinx formation in patients 10 years post administration of the OPC1 product.^125^ This data set provides solid evidence supporting the safety of OPC1 with an event-free period of up to 10 years, which strengthens the safety profile of the SCiStar trial.

Analysis of the global trends in clinical trials using hPSC-based therapy showed that 77.1% of studies were observational (no cells were administered into patient), and only 22.9% of studies used hPSC-derived cells as interventional treatment.^126^ The number of studies using hiPSCs was relatively higher than that using hESCs, which was 74.8% compared to 25.2%, respectively. The majority of observational studies were performed in developed countries, including the USA (41.6%) and France (16.8%), whereas interventional studies were conducted in Asian countries, including China (36.7%), Japan (13.3%), and South Korea (10%). The trends in therapeutic studies were also clear in terms of targeted diseases. The three most studied diseases were ophthalmological conditions, circulatory disorders, and nervous systems.^127^ However, it is surprising that the clinical applications of hPSCs have achieved little progress since the first hESCs were discovered worldwide. The relatively low number of clinical trials focusing on using iPSCs as therapeutic agents to administer into patients could be ascribed to the unstable genome of hiPSCs,^128^ immunological rejection,^129^ and the potential for tumor formation.^130^

## Mesenchymal stem/stromal cell-based therapy: is it time to consider their origin toward targeted therapy?

Approximately 55 years ago, fibroblast-like, plastic-adherent cells, later named mesenchymal stem cells (MSCs) by Arnold L. Caplan,^18^ were discovered for the first time in mouse bone marrow (BM) and were later demonstrated to be able to form colony-like structures, proliferate, and differentiate into bone/reticular tissue, cartilage, and fat.^131^ Protocols were subsequently established to directly culture this subpopulation of stromal cells from BM in vitro and to stimulate their differentiation into adipocytes, chondroblasts, and osteoblasts.^132^ Since then, MSCs have been found in and derived from different human tissue sources, including adipose tissue (AT), the umbilical cord (UC), UC blood, the placenta, dental pulp, amniotic fluid, etc.^133^ To standardize and define MSCs, the International Society for Cell and Gene Therapy (ISCT) set minimal identification criteria for MSCs derived from multiple tissue sources.^134^ Among them, MSCs derived from AT, BM, and UC are the most commonly studied MSCs in human clinical trials,^135^ and they constitute the three major tissue sources of MSCs that will be discussed in this review.

The discovery of MSCs opened an era during which preclinical studies and clinical trials have been performed to assess the safety and efficacy of MSCs in the treatment of various diseases. The major conclusion of these studies and trials is that MSC-based therapy is safe, although the outcomes have usually been either neutral or at best marginally positive in terms of the clinically relevant endpoints regardless of MSC tissue origin, route of infusion, dose, administration duration, and preconditioning.^136^ It is important to note that a solid background of knowledge has been generated from all these studies that has fueled the recent translational research in MSC-based therapy. As MSCs have been intensively studied over the last 55 years and have become the subject of multiple reviews, systematic reviews, and meta-analyses, the objective of this paper is not to duplicate these publications. Rather, we will discuss the questions that both clinicians and researchers are currently exploring with regard to MSC-based therapy, diligently seeking answers to the following:

- “With a solid body of data supporting their safety profiles derived from both preclinical and clinical studies, does the tissue origin of MSCs also play a role in their downstream clinical applications in the treatment of different human diseases?”
- “Do MSCs derived from AT, BM, and UC exhibit similar efficacy in the treatment of neurological diseases, metabolic/endocrine-related disorders, reproductive dysfunction, skin burns, lung fibrosis, pulmonary disease, and cardiovascular conditions?”

To answer these questions, we will first focus on the most recently published clinical data regarding these targeted conditions, including neurological disorders, pulmonary dysfunctions, metabolic/endocrine-related diseases, reproductive disorders, skin burns, and heart-related diseases, to analyze the potential efficacy of MSCs derived from AT, BM, and UC. Based on the level of clinical improvement observed in each trial, we analyzed the potential efficacy of MSCs derived from each source to visualize the correlation between patient improvement and MSC sources. We will then address recent trends in the exclusive use of MSC-based products, focusing on the efficacy of treatment with MSCs from each of the abovementioned sources, and we will analyze the relationship between the respective efficacies of MSCs from these sources in relation to the targeted disease conditions. Finally, we propose a hypothesis and mechanism to achieve the currently still unmet objective of evaluating the use of MSCs from AT, BM, and UC in regenerative medicine.

## An overview of MSC tissue origins and therapeutic potential

**Fig. 2:**
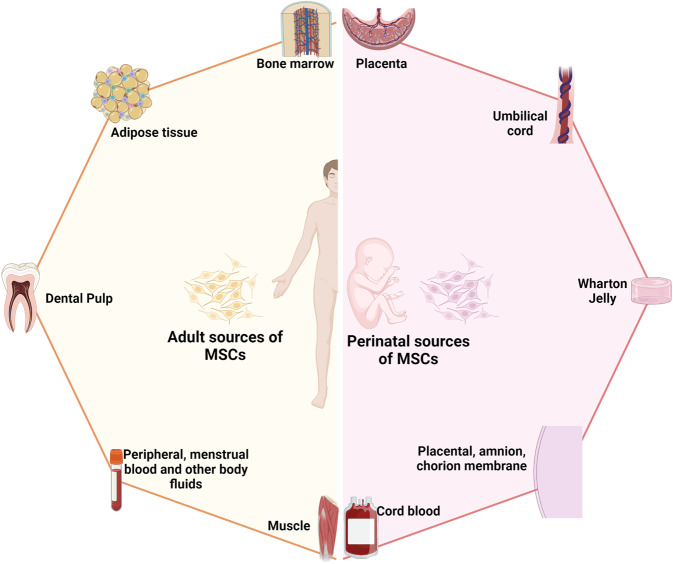
The two major sources of MSCs: adult and perinatal sources. The adult sources of MSCs are specific tissue in human body where MSCs could be isolated, including bone marrow, adipose tissue, dental pulp, peripheral blood, menstrual blood, muscle, etc. The perinatal sources of MSCs consist of umbilical cord-derived components, such as umbilical cord, Wharton’s jelly, umbilical cord blood, and placental structures, such as placental membrane, amnion, chorion membrane, amniotic fluid, etc. The figure was created with BioRender.com

In general, MSCs are reported to be isolated from numerous tissue types, but all of these types can be organized into two major sources: adult^137^ and perinatal sources^138^ (Fig. 2). Adult sources of MSCs are defined as tissues that can be harvested or obtained from an individual, such as dental pulp,^139^ BM, peripheral blood,^140^ AT,^141^ lungs,^142^ hair,^143^ or the heart.^144^ Adult MSCs usually reside in specialized structures called stem cell niches, which provide the microenvironment, growth factors, cell-to-cell contacts and external signals necessary for maintaining stemness and differentiation ability.^145^ BM was the first adult source of MSCs discovered by Friedenstein^131^ and has become one of the most documented and largely used MSC sources to date, followed by AT. BM-MSCs are isolated and cultured in vitro from BM aspirates using a Ficoll gradient-centrifugation method^146^ or a red blood cell lysate buffer to collect BM mononuclear cell populations, whereas AT-MSCs are obtained from stromal vascular fractions of enzymatically digested AT obtained through liposuction,^141^ lipoplasty, or lipectomy procedures.^147^ These tissue collection procedures are invasive and painful for the patient and are accompanied by a risk of infection, although BM aspiration and adipose liposuction are considered safe procedures for BM and AT biopsies. The number of MSCs that can be isolated from these adult tissues varies significantly in a tissue-dependent manner. The percentage of MSCs in BM mononuclear cells ranges from 0.001 to 0.01% following gradient centrifugation.^132^ The number of MSCs in AT is at least 500 times higher than that in BM, with approximately 5,000 MSCs per 1 g of AT. Perinatal sources of MSCs consist of UC-derived components, such as UC, Wharton’s jelly, and UC blood, and placental structures, such as the placental membrane, amnion, chorion membrane, and amniotic fluid.^138^ The collection of perinatal MSCs, such as UC-MSCs, is noninvasive, as the placenta, UC, UC blood, and amnion are considered waste products that are usually discarded after birth (with no ethical barriers).^148^ Although MSCs represent only 10^−7^% the cells found in UC, their higher proliferation rate and rapid population doubling time allow these cells to rapidly replicate and increase in number during in vitro culture.^149^ Under standardized xeno-free and serum-free culture platforms, AT-MSCs show a faster proliferation rate and a higher number of colony-forming units than BM-MSCs.^149^ UC-MSCs have the fastest population doubling time compared to AT-MSCs and BM-MSCs in both conventional culture conditions and xeno- and serum-free environments.^149^ MSCs extracted from AT, BM and UC exhibit all minimal criteria listed by the ISCT, including morphology (plastic adherence and spindle shape), MSC surface markers (95% positive for CD73, CD90 and CD105; less than 2% negative for CD11, CD13, CD19, CD34, CD45, and HLR-DR) and differentiation ability into chondrocytes, osteocytes, and adipocytes.^150^

In fact, although MSCs derived from either adult or perinatal sources exhibit similar morphology and the basic characteristics of MSCs, studies have demonstrated that these cells also differ from each other. Regarding immunophenotyping, AT-MSCs express high levels of CD49d and low levels of Stro-1. An analysis of the expression of CD49d and CD106 showed that the former is strongly expressed in AT-MSCs, in contrast to BM-MSCs, whereas CD106 is expressed in BM-MSCs but not in AT-MSCs.^151^ Increased expression of CD133, which is associated with stem cell regeneration, differentiation, and metabolic functions,^152^ was observed in BM-MSCs compared to MSCs from other sources.^153^ A recent study showed that CD146 expression in UC-MSCs was higher than that in AT- and BM-MSCs,^153^ supporting the observation that UC-MSCs have a stronger attachment and a higher proliferation rate than MSCs from other sources, as CD146 is a key cell adhesion protein in vascular and endothelial cell types.^154^ In terms of differentiation ability, donor-matched BM-MSCs exhibit a higher ability to differentiate into chondrogenic and osteogenic cell types than AT-MSCs, whereas AT-MSCs show a stronger capacity toward the adipogenic lineage.^150^ The findings from an in vitro differentiation study indicated that BM-MSCs are prone to osteogenic differentiation, whereas AT-MSCs possess stronger adipogenic differentiation ability, which can be explained by the fact that the epigenetic memory obtained from either BM or AT drives the favored MSC differentiation along an osteoblastic or adipocytic lineage.^155^ Interestingly, although UC-MSCs have the ability to differentiate into adipocytes, osteocytes, or chondrocytes, their osteogenic differentiation ability has been proven to be stronger than that of BM-MSCs.^156^ The most interesting characteristic of MSCs is their immunoregulatory functions, which are speculated to be related to either cell-to-cell contact or growth factor and cytokine secretion in response to environmental/microenvironmental stimuli. MSCs from different sources almost completely inhibit the proliferation of peripheral blood mononuclear cells (PBMCs) at PBMC:MSC ratios of 1:1 and 10:1.^149^ At a higher ratio, BM-MSCs showed a significantly higher inhibitory effect than AT- or UC-MSCs.^153^ Direct analysis of the immunosuppressive effects of BM- and UC-MSCs has revealed that these cells exert similar inhibitory effects in vitro with different mechanisms involved.^157^ With these conflicting data, the mechanism of action related to the immune response of MSCs from different sources is still poorly understood, and long-term investigations both in preclinical studies and in clinical trial settings are needed to shed light on this complex immunomodulation function.

The great concern in MSC-based therapy is the fate of these cells post administration, especially through different delivery routes, including systemic administration via an intravenous (IV) route or tissue-specific administration, such as dorsal pancreatic administration. It is important to understand the distribution of these cells after injection to expand our understanding of the underlying mechanisms of action of treatments; in addition, this knowledge is required by authorized bodies (the Food and Drug Administration (FDA) in the United States or the regulation of advanced-therapy medicinal products in Europe, No. 1394/2007) prior to using these cells in clinical trials. The preclinical data using various labeling techniques provide important information demonstrating that MSCs do not have unwanted homing that could lead to the incorrect differentiation of the cells or inappropriate tumor formation. In a mouse model, human BM-MSCs and AT-MSCs delivered via an IV route are rapidly trapped in the lungs and then recirculate through the body after the first embolization process, with a small number of infused cells found mainly in the liver after the second embolization.^158^ Using the technetium-99 m labeling method, intravenously infused human cells showed long-term persistence up to 13 months in the bone, BM compartment, spleen, muscle, and cartilage.^159^ A similar result was reported in baboons, confirming the long-term homing of human MSCs in various tissues post administration.^160^ Although the retainment of MSCs in the lungs might potentially reduce their systemic therapeutic effects,^161^ it provides a strong advantage when these cells are used in the treatment of respiratory diseases. Local injection of MSCs also revealed their tissue-specific homing, as an injection of MSCs via the renal artery route resulted in the majority of the injected cells being found in the renal cortex.^162^ Numerous studies have been conducted to track the migration of administered MSCs in human subjects. Henriksson and his team used MSCs labeled with iron sucrose in the treatment of intervertebral disc degeneration.^163^ Their study showed that chondrocytes differentiated from infused MSCs could be detected at the injured intervertebral discs at 8 months but not at 28 months. A study conducted in a patient with hemophilia A using In-oxine-labeled MSCs showed that the majority of the cells were trapped in the lungs and liver 1 h post administration, followed by a reduction in the lungs and an increase in the number of cells in the liver after 6 days.^164^ Interestingly, a small proportion of infused MSCs were found in the hemarthrosis site at the right ankle after 24 h, suggesting that MSCs are attracted and migrate to the injured site. The distribution of MSCs was also reported in the treatment of 21 patients diagnosed with type 2 diabetes using 18-FDG-tagged MSCs and visualized using positron emission tomography (PET).^165^ The results illustrated that local delivery of MSCs via an intraarterial route is more effective than delivery via an IV route, as MSCs home to the pancreatic head (pancreaticoduodenal artery) or body (splenic artery). Therefore, although the available data related to the biodistribution of infused MSCs are still limited, the results obtained from both preclinical and clinical studies illustrate a comparable set of data supporting results on homing, migration to the injured site, and the major organs where infused MSCs are located. The following comprehensive and interesting reviews are highly recommended.^166–168^

To date, 1426 registered clinical trials spanning different trial phases have used MSCs for therapeutic purposes, which is four times the number reported in 2013.^169,170^ As supported by a large body of preclinical studies and advancements in conducting clinical trials, MSCs have been proven to be effective in the treatment of numerous diseases, including nervous system and brain disorders, pulmonary diseases,^171^ cardiovascular conditions,^172^ wound healing, etc. The outcomes of MSC-based therapy have been the subject of many intensive reviews and systematic analyses with the solid conclusion that these cells exhibit strong safety profiles and positive outcomes in most tested conditions.^173–175^ In addition, the available data have revealed several potential mechanisms that could explain the beneficial effects of MSCs, including their homing efficiency, differentiation potential, production of trophic factors (including cytokines, chemokines, and growth factors), and immunomodulatory abilities. However, it is still not known which MSC types should be used for which diseases, as it seems to be that MSCs exhibit beneficial effects regardless of their sources.^169^

## Acquired brain and spinal cord injury treatment: BM-MSCs have emerged as key players

The theory that brain cells can never regenerate has been challenged by the discovery of newly formed neurons in the human adult hippocampus or the migration of stem cells in the brain in animal models.^176^ These observations have triggered hope for regeneration in the context of neuronal diseases by using exogenous stem cell sources to replenish or boost the stem cell population in the brain. Moreover, the limited regenerative capacity of the brain and spinal cord is an obstacle for traditional treatments of neurodegenerative diseases, such as autism, cerebral palsy, stroke, and spinal cord injury (SCI). As current treatments cannot halt the progression of these diseases, studies throughout the world have sought to exploit cell-based therapies to treat neurodegenerative diseases on the basis of advances in the understanding and development of stem cell technology, including the use of MSCs. Successful stem cell therapy for treating brain disease requires therapeutic cells to reach the injured sites, where they can repair, replace, or at least prevent the deteriorative effects of neuronal damage.^177^ Hence, the gold standard of cell-based therapy is to deliver the cells to the target site, stimulate the tissue repair machinery, and regulate immunological responses via either cell-to-cell contact or paracrine effects.^178^ Among 315 registered clinical trials using stem cells for the treatment of brain diseases, MSCs and hematopoietic stem cells (HSCs; CD34+ cells isolated from either BM aspirate or UC blood) are the two main cell types investigated, whereas approximately 102 clinical trials used MSCs and 62 trials used HSCs for the treatment of brain disease (main search data from clinicaltrial.gov). MSCs are widely used in almost all clinical trials targeting different neuronal diseases, including multiple sclerosis,^179^ stroke,^180^ SCI,^181^ cerebral palsy,^182^ hypoxic-ischemic encephalopathy,^183^ autism,^184^ Parkinson’s disease,^185^ Alzheimer’s disease^185^ and ataxia. Among these trials in which MSCs were the major cells used, nearly two-thirds were for stroke, SCI, or multiple sclerosis. MSCs have been widely used in 29 registered clinical trials for stroke, with BM-MSCs being used in 16 of these trials. With 26 registered clinical trials, SCI is the second most common indication for using MSCs, with 16 of these trials using mainly expanded BM-MSCs. For multiple sclerosis, 15 trials employed BM-MSCs among a total of 23 trials conducted for the treatment of this disease. Hence, it is important to note that in neuronal diseases and disorders, BM-MSCs have emerged as the most commonly used therapeutic cells among other MSCs, such as AT-MSCs and UC-MSCs.

The outcomes of the use of BM-MSCs in the treatment of neuronal diseases have been widely reported in various clinical trial types. A review by Zheng et al. indicated that although the treatments appeared to be safe in patients diagnosed with stroke, there is a need for well-designed phase II multicentre studies to confirm the outcomes.^173^ One of the earliest trials using autologous BM-MSCs was conducted by Bang et al. in five patients diagnosed with stroke in 2005. The results supported the safety and showed an improved Barthel index (BI) in MSC-treated patients.^186^ In a 2-year follow-up clinical trial, 16 patients with stroke received BM-MSC infusions, and the results showed that the treatment was safe and improved clinical outcomes, such as motor impairment scale scores.^187^ A study conducted in 12 patients with ischemic stroke showed that autologous BM-MSCs expanded in vitro using autologous serum improved the patient’s modified Rankin Scale (mRS), with a mean lesion volume reduced by 20% at 1 week post cell infusion.^188^ In 2011, a modest increase in the Fugl Meyer and modified BI scores was observed after autologous administration of BM-MSCs in patients with chronic stroke.^189^ More recently, a prospective, open-label, randomized controlled trial with blinded outcome evaluation was conducted, with 39 patients and 15 patients in the BM-MSC administration and control groups, respectively. The results of this study indicated that autologous BM-MSCs with autologous serum administration were safe, but the treatment led to no improvements at 3 months in modified Rankin Scale (mRS) scores, although leg motor improvement was observed.^180^ Researchers explored whether the efficacy of BM-MSC administration was maintained over time in a 5-year follow-up clinical trial. Patients (85) were randomly assigned to either the MSC group or the control group, and follow-ups on safety and efficacy were performed for 5 years, with 52 patients being examined at the end of the study. The MSC group exhibited a significant improvement in terms of decreased mRS scores, whereas the number of patients with an mRS score increase of 0–3 was statistically significant.^187^ Although autologous BM-MSCs did not improve the Basel index, mRS, or National Institutes of Health Stroke Scale (NIHSS) score 2 years post infusion, patients who received BM-MSC therapy showed improvement in their motor function score.^190^ In addition, a prospective, open-label, randomized controlled trial by Lee et al. showed that autologous BM-MSCs primed with autologous “ischemic” serum significantly improved motor functions in the MSC-treated group. Neuroimaging analysis also illustrated a significant increase in interhemispheric connectivity and ipsilesional connectivity in the MSC group.^191^ Recently, a single intravenous infection of allogeneic BM-MSCs has been proven to be safe and feasible in patients with chronic stroke with a significant improvement in BI score and NIHSS score.^192^

In two systematic reviews using MSCs for the treatment of SCI, BM-MSCs (*n* = 16) and UC-MSCs (*n* = 5) were reported to be safe and well-tolerated.^193,194^ The results indicated significant improvements in the stem cell administration groups compared with the control groups in terms of a composite of the American Spinal Injury Association (ASIA) impairment scale (AIS) grade, AIS grade A, and ASIA sensory scores and bladder function (Table 1). However, larger experimental groups with a randomized and multicentre design are needed for further confirmation of these findings. For multiple sclerosis, several early-phase (phase I/II) registered clinical studies have used BM-MSCs. A study compared the potential efficacy of BM-MSC and BM mononuclear cell (BMMNC) transplantation in 105 patients with spastic cerebral palsy.^195^ The results showed that the GMFM (gross motor function measure) and the FMFM (fine motor function measure) scores of the BM-MSC transplant group were higher than those of the BMNNC transplant group at 3, 6, and 12 months of assessment. In terms of autism spectrum disorder, a review of 254 children after BMMNC transplantation found that over 90% of patients’ ISAA (Indian Scale for Assessment of Autism) and CARS (Childhood Autism Rating Scale) scores improved. Young patients and those in whom autism spectrum disorder was detected early generally showed better improvement.^196^

**Table 1:** The reported clinical trials using MSCs from AT, BM, and UC in the treatment of brain-related injuries and neurological disorders

Year	Disease	MSC source	No. of MSC-treated patients	Efficacy
2022^206^	Acute ischemic stroke	AT-MSC	4	No significant improvement compared to placebo in mRS and NIHSS score.
2014^461^	Acute ischemic stroke	AT-MSC	10	Potential efficacy of intravenous administration of allogeneic AT-MSCs within the first 2 weeks of stroke.
2020^196^	Autism spectrum disorders	BM-MSC	254	After transplantation, 94.48% patients showed a positive change on ISAA (Indian Scale for Assessment of Autism) and 95.27% of patients showed an improved score on CARS (Childhood Autism Rating Scale) and 86 (86/86) patients showed improved brain activity through the FDG-PET CT scan
2020^201^	Autism spectrum disorders	UC-MSC	12	Six of 12 participants demonstrated improvement in at least two ASD-specific measures
2017^195^	Cerebral palsy	BM-MSC	35	Scores of A, B, C, D, E and total scores of GMFM and FMFM significant improvement compared to before transplantation and control group
2020^199^	Cerebral palsy	UC-MSC	19	The ADL, CFA, and GMFM-88 scores significant improvement compared to before transplantation and control group
2011^189^	Chronic stroke	BM-MSCs	12	A modest increase in Fugl Meyer and modified Barthel index score.
				Increase the number of cluster activation of Brodmann areas 4 and 6 after MSC infusion.
2019^192^	Chronic stroke	Allogeneic BM-MSCs	36	The treatment was safe and well-tolerated based on serial exams, electrograms, laboratory tests, and computed tomography scans of chest/abdomen/pelvis.
				All behavioral endpoints showed significant improvement over 12 months of follow-up.
2005^186^	Ischemic stroke	BM-MSCs	5	Improve motor functions in the MSC-treated group during the follow-up period with no statistical significance.
2010^187^	Ischemic stroke	BM-MSC	16	mRS score significant improvement over the control group
2011^188^	Ischemic stroke	BM-MSCs	12	Slight change in NIHSS and mean lesion volume after the first week of infusion.
				Slight improvement in mRS score.
2021^180^	Ischemic stroke	BM-MSC	39	lower extremity motor function significant improvement over the control group
2020^190^	Ischemic stroke	BM-MSC	16	Did not improve the Basel index, mRS, and NIHSS after 2 years post infusion.
				MSC-based therapy might improve motor performance and task-related primary motor cortex activity.
2022^191^	Ischemic stroke	BM-MSC	31	Significant improvement in motor functions in MSC group.
				In neuroimaging analysis, corticospinal tract and posterior limb of the internal capsule fractional anisotrophy did not reduced in the MSC group but significantly decreased in the control group 90 days post infusion.
				Interhemispheric connectivity and ipsilesional connectivity significantly increased in the MSC group.
2018^462^	Ischemic stroke	Allogeneic UC-MSC	10	A slight improvement in mRS and NIHSS score relative to baseline.
2013^203^	Spinal cord injury	UC-MSC	22	Treatment was effective in 13 of 22 patients in ASIA, and IANR-SCIFRS scores
2021^202^	Spinal cord injury	UC-MSC	41	Significant improvement compared to before transplantation in ASIA total score, pinprick score and light touch, IANR-SCIFRS total score and sphincter score
2009^463^	Spinal cord injury	BM-MSC	10	Improvement in ASIA score, SEP and EMG.
2012^464^	Spinal cord injury	BM-MSC	10	6/10 patients showed improvement of motor power of the upper extremities at a 6-month follow-up
				3/10 patients showed gradual improvement in activities of daily living.
				MRI showed reduction in cavity size and the presence of fiber-like slow signal intensity steaks.
2012^465^	Spinal cord injury	BM-MSC	5	Significant improvement was observed in patients with AIS grade B and C.
2013^466^	Spinal cord injury	BM-MSC	50	BM-MSC-treated patients combined with physical therapy showed functional improvement over the control group.
				At 18-month follow-up, 23/50 MSC-treated cases (46%) maintained functional improvement.
2012^467^	Spinal cord injury	BM-MSC	11	5/11 (45%) MSC-infused cases showed remarkable recovery in AIS grade from A to C compared to 3/20 (15%) patients in the control group.
2009^468^	Spinal cord injury	BM-MSC	30	No changes in AIS grade.
				No significant changes in SEP, MEP, and NCV.
2014^469^	Spinal cord injury	BM-MSC	14	7 MSC-infused patients improved AIS score.
2018^469,470^	Spinal cord injury	BM-MSC	11	10 patients showed improvement in their neurological dysfunction after MSC infusion, although there was no statistical significance.
2013^471^	Spinal cord injury	BM-MSC	20	The clinical symptoms were improved in 10 patients with total effective rate 50% (improved AIS grade A, B, and C).
				BM-MSCs can effectively improve the neurologic dysfunction associated with complete and chronic cervical spinal cord injury.
2013^472^	Spinal cord injury	BM-MSC	20	30 days post administration, 15 patients showed improvement, including 4/8 patients with SCI grade A, 3/4 patients with SCI grade B, and 8 patients with SCI grade C.
2015^473^	Spinal cord injury	BM-MSC	1	Improvement in AIS grade.
2018^474^	Cerebral palsy	UC-MSC	27	The significant improvement compared to the control group in GMFM-88, CFA score
2010^475^	Multiple sclerosis	BM-MSC	15	- EDSS score improved from 6.7 (1.0) to 5.9 (1.6).
				- 24 h after MSC transplantation: increase in the proportion of CD4+CD25+ regulatory T cells, a decrease in the proliferative responses of lymphocytes, and the expression of CD40+, CD83+, CD86+, and HLA-DR on myeloid dendritic cells
2010^476^	Multiple sclerosis	BM-MSC	10	- The EDSS improvement in 5/7 patients
				- Vision and low contrast sensitivity testing at 3 months showed improvement in 5/6
2011^477^	Multiple sclerosis	BM-MSC	7	The expression of the FOXP3 was significantly increased compared to before transplantation (*P*<0.005)
2011^478^	Multiple sclerosis	BM-MSC	8	The improvement of 0.5-1 point on EDSS was seen in 6/8, stabilization in 1/8, progression in 1/8
2012^479^ 2013^480^	Multiple sclerosis	BM-MSC	25	- No statistically significant variations in gene expression and serum level of cytokines after a 1-year follow-up of MSC-treated MS patients
				- No significant improvement compared to before transplantation in EDSS score and MRI evaluation
2014^481^	Multiple sclerosis	BM-MSC	9	GEL had a trend to lower mean cumulative number
2016^482^	Multiple sclerosis	BM-MSC	6	4/6 patients showed a measurable clinical improvement following MSC-NP treatment.
2017^483^	Multiple sclerosis	BM-MSC	10	The overall trend of improvement in EDSS and secondary clinical test included (25 WFT, 9-PHT), cognitive (MMSE), and ophthalmology (OCT, VEP)
2018^484^	Multiple sclerosis	UC-MSC	2	Improvement of physical and mental problems in both patients
2018^485^	Multiple sclerosis	AD-MSC	34	Measures of treatment effect showed an inconclusive trend of efficacy include EDSS and magnetic resonance-imaging

*25 WFT* Timed 25‐Foot Walk, *9-PHT* 9 Hole Peg Test, *ADL* Activities of Daily Living, *AIS* American Spinal Cord Injury Association (ASIA) Impairment Scale, *CARS* Childhood Autism Rating Scale *CFA* Comprehensive functional assessment, EDSS The Expanded Disability Status Scale, *EMG* electromyography, *FMFM* Fine motor function measurement, *FOXP3* forkhead box P3, also known as scurfin, *GMFM* Gross motor function measurement, *GMFM-88* Gross motor function measurement-88, *IANR-SCIFRS* International Association of Neurorestoratology-Spinal Cord Injury Functional Rating Scale, *ISAA* Indian Scale for Assessment of Autism, *MMSE* Mini‐Mental Status Examination, *MRI* Magnetic Resonance Imaging, *mRS* modified Rankin Scale, *NCV* nerve conduction velocity, *NIHSS* National Institutes of Health Stroke Scale, *OCT* Optical Coherence Tomography, *SCI* Spinal Cord Injury, *SER* somatosensory evoked potentials, *VEP* Visual Evoked Potential

One of the biggest limitations when using BM-MSCs is the bone marrow aspiration process, as it is an invasive procedure that can introduce a risk of complications, especially in pediatric and elderly patients.^197^ Therefore, UC-MSCs have been suggested as an alternative to BM-MSCs and are being studied in clinical trials for the treatment of neurological diseases in approximately 1550 patients throughout the world; however, only three studies have been completed, with data published recently.^198^ A recent study showed that UC-MSC administration improved both gross motor function and cognitive skills, assessed using the Activities of Daily Living (ADL), Comprehensive Function Assessment (CFA), and GMFM, in patients diagnosed with cerebral palsy. The improvements peaked 6 months post administration and lasted for 12 months after the first transplantation.^199^ In a single-targeted phase I/II clinical trial using UC-MSCs for the treatment of autism, Riordan et al. reported decreases in Autism Treatment Evaluation Checklist (ATEC) and CARS scores for eight patients, but the paper has been retracted due to a violation of the journal’s guidelines.^200^ In an open-label, phase I study, UC-MSCs were used as the main cells to treat 12 patients with autism spectrum disorder via IV infusions. It is important to note that five participants developed new class I anti-human leukocyte antigen in response to the specific lot of manufactured UC-MSCs, although these responses did not exhibit any immunological response or clinical manifestations. Only 50% of participants showed improvements in at least two autism-specific measurements.^201^ Although not as widely used as BM-MSCs, these trials have demonstrated the efficacy of using UC-MSCs in the treatment of SCIs. In a pilot clinical study, Yang et al. showed that the use of UC-MSCs has the potential to improve disease status through an increase in total ASIA and SCI Functional Rating Scale of the International Association of Neurorestoratology (IANR-SCIFRS) scores, as well as an improvement in pinprick, light touch, motor and sphincter scores.^202^ A study of 22 patients with SCIs showed a potential therapeutic effect in 13 patients post UC-MSC infusion.^203^ AT-MSCs were also used to treat SCI, with a single case report indicating an improvement in neurological and motor functions in a domestic ferret patient.^204^ However, a result obtained from another phase I trial using AT-MSCs showed mild improvements in neurological function in a small number of patients.^205^ A phase II, randomized, double-blind, placebo-controlled, single-center, pilot clinical trial using AT-MSCs in the treatment of acute ischemic stroke published a data set that supports the safety of the therapy, although patients who received AT-MSCs showed a nonsignificant improvement after 24 months of follow-up.^206^ In all of the above studies, the safety of using either AT-MSCs or UC-MSCs was evaluated, and no significant reactions were reported after infusion.

Therefore, based on the number of recovered patients post-transplantation and the number of recruited patients in large-scale trials using BM-MSCs, it seems that BM-MSCs are the prominent cells in regard to treating neurodegenerative disease with potentially good outcomes (Table 1). It is important to note that we do not negate the fact that AT- and UC-MSCs also show positive outcomes in the treatment of neuronal diseases, with numerous ongoing large-scale, multicentre, randomized, and placebo-control trials,^207,208^ but we suggest alternative and thoughtful decisions regarding which sources of MSCs are best for the treatment of neuronal diseases and degenerative disorders.

## Respiratory disease and lung fibrosis: clinical data support UC as a good source of MSCs

In the last decade, significant increases in respiratory disease incidence due to air pollution, smoking behavior, population aging, and recently, respiratory virus infections such as coronavirus disease 2019 (COVID-19)^209^ have been observed, leading to substantial burdens on public health and healthcare systems worldwide. Respiratory inflammatory diseases, including bronchopulmonary dysplasia (BPD), chronic obstructive pulmonary disease (COPD), and acute respiratory distress syndrome (ARDS), have recently emerged as three prevalent pulmonary diseases in children and adults. These conditions are usually associated with inflammatory cell infiltration, a disruption of alveolar structural integrity, a reduction in alveolar fluid clearance ability, cytokine release and associated cytokine storms, airway remodeling, and the development of pulmonary fibrosis. Traditional treatments are focused on relieving symptoms and preventing disease progression using surfactants, artificial respiratory support, mechanical ventilation, and antibiotic/anti-inflammatory drugs, with limited effects on the damaged airway, alveolar fluid clearance, and other detrimental effects caused by the inflammatory response. MSCs are known for their immunomodulatory abilities, showing potential in injury reduction and aiding lung recovery after injury. According to ClinicalTrials.gov, from 2017 to date, there have been 159 studies testing the application of MSCs in the treatment of pulmonary diseases, including but not limited to BPD, COPD, and ARDS, suggesting a trend in the use of MSCs as an alternative approach for the treatment of respiratory diseases, especially MSCs from UC as an “off-the-shelf” and allogeneic source.

Extremely premature infants are born with arrested lung development at the canalicular-saccular phases prior to alveolarization and before pulmonary maturation occurs, which results in the development of BPD.^210^ These infants require intensive care during the first three months of life using postnatal interventions, including positive pressure mechanical ventilation, external oxygen support, and surfactant infusions, and the newborns have recurrent infections that further compromise normal lung development.^211^ To date, 13 clinical trials have been proposed to use UC-MSCs in the treatment of BPD, recruiting ~566 premature infants throughout the world, including Vietnam, Korea, the United States, Spain, Australia, and China. The majority of these trials use UC-derived stem cells for phases I and II, focusing on evaluating the safety and efficacy of stem cell-based therapy.^212^ Human UC tissue and its derivative components are considered the most attractive cell sources for MSCs in the treatment of BPD due to the ease of obtaining them, being readily available, with no ethical concerns, low antigenicity, a high cell proliferation rate, and superior regenerative potential. Chang et al. used MSCs derived from UC blood in a phase I dose-escalation clinical trial to treat 9 preterm infants via intratracheal administration to prevent the development of BPD.^213^ All 9 preterm infants survived, and only three developed BPD; these infants had significantly decreased BPD severity compared with the historically matched control group. A follow-up study of the same patients after 24 months indicated that only one infant had an *E. cloacae* infection after discharge at 4 months, with subsequent disseminated intravascular coagulation, which was later proven to be unrelated to the intervention. The remaining eight patients survived with normal pulmonary development and function, suggesting that the therapy was safe. MSCs from UC blood were also used for the treatment of 12 extremely low birthweight preterm patients using the same administration route, which further confirmed the safety of the therapy in the treatment of BPD, although ten of 12 infants still developed severe BPD at 36 weeks.^214^ Our group also reported the safety and potential efficacy of using UC-MSCs in the treatment of four preterm infants, and the results supported the safety of UC-MSCs and demonstrated that patients could be weaned from oxygen supply and develop normal lung structure and function.^215^ A phase II clinical trial of 66 infants born at 23–28 weeks with a birthweight of 500–1250 g who were recruited and randomized into an MSC-administration group and a control group was conducted. Although the results supported the safety of MSC administration in preterm infants, the efficacy of the treatment was not supported by statistical analysis, potentially due to the small sample size. Subgroup analysis showed that patients with severe BPD born at 23–24 weeks showed a significant improvement in BPD severity, but those born at 25–28 weeks did not.^216^ Hence, it is important to conduct controlled phase II clinical trials with larger cohort sizes to further substantiate the efficacy of UC blood-derived MSCs in the treatment of infants with BPD.

With more than 65 million patients worldwide, COPD was the third-leading cause of death in 2020, according to World Health Organization records. COPD is classified as a chronic inflammatory and destructive pulmonary disease characterized by a progressive reduction in lung function. Averyanov et al. performed a randomized, placebo-controlled phase I/IIa study in 20 patients with mild to moderate idiopathic pulmonary fibrosis (IPF). Treatment group patients received two IV doses of allogeneic MSCs (2 × 10^8^ cells) every 3 months, and the second group received a placebo.^217^ Evaluation tests were performed at weeks 13, 26, 39, and 52. The 6-min walking test distance (6MWTD) results showed that patient fitness improved from week 13 onwards and was maintained until up to the 52nd week. Pulmonary function indicators improved markedly before and after treatment in the treated group but did not change significantly in the placebo group. The goal of MSC therapy in the treatment of COPD is to promote the regeneration of parenchymal cells and alveolar structure and the restoration of lung function. Based on the results of a phase I trial of a commercial BM-MSC product, Prochymal^TM^, which led to improvements in pulmonary function in treated patients, a multicentre, double-blind, placebo-controlled phase II trial was conducted in 62 patients diagnosed with COPD to determine the safety and potential efficacy of the product. Although the results supported the safety of BM-MSCs, their effectiveness in the treatment of COPD was not assured. No statistically significant differences in FEV_1_ or FEV_1%_, total lung capacity, or carbon monoxide diffusing capacity were detected after 2 years of follow-up between the two treatment groups. To date, there have been five clinical trials using BM-MSCs as the main stem cells for the treatment of COPD, but the overall clinical outcomes did not demonstrate the potential therapeutic effects of the treatment.^218–222^ In clinical trial NCT001110252, the results showed that there was an overall reduction in the process of COPD pathological development 3 years after the administration of BM-MSCs, although the trial had a phase I design, with no control group, and evaluated only a small cohort (four patients).^219^ To alleviate local inflammatory progression in COPD, Oliveira et al. studied the combination treatment of one-way endobronchial valve (EBV) and BM-MSC intubation.^223^ Ten GOLD (Global Initiative for Obstructive Lung Disease) stage C or D patients were equally divided into 2 groups: one group received a dose of 10^8^ cells before valve insertion, and the other group received a normal saline infusion. The follow-up time was 90 days. Inflammation was significantly improved as assessed by the CRP (C-reactive protein) index at 30 and 90 days after infusion. In addition, improvements in St. George’s Respiratory Questionnaire (SGRQ) scores indicated improved patient quality of life. Furthermore, an investigation into the homing ability of MSCs in vivo was performed on 9 GOLD patients, from stage A to stage D. Each patient received two 2 × 10^6^ BM-MSC/kg IV infusions 1-week apart.^224^ The marking of MSCs with indium-111 showed that MSCs were retained in the pulmonary vasculature longer in patients with mild COPD and that the levels of inflammatory mediators improved after 7 days of treatment. The results of the evaluation survey conducted after 1 year showed that the number of COPD exacerbations decreased to six times/year compared to 11 times/year before treatment. In addition, AT-MSCs present in the stromal vascular fraction were used to treat patients with COPD, and no adverse events were observed after 12 months of follow-up, but the clinical improvements post administration were not clear.^225^ The results from a phase I clinical trial using AT-MSCs in eight patients with COPD also reported no significant change in pulmonary function test parameters.^226^ A study evaluating the use of AT-MSCs as adjunctive therapy for COPD in 12 patients was performed.^227^ AT was obtained using standard liposuction, MSCs were isolated, and 150–300 million cells were intravenously infused. The patients showed improvements in quality of life, with improved SGRQ scores after 3 and 6 months of treatment. Recently, UC-MSCs have emerged as potential allogeneic stem cell candidates for the treatment of COPD.^228^ In a pilot clinical study, it was demonstrated that allogeneic administration of UC-MSCs in the treatment of COPD was safe and potentially effective.^229^ In one study, 20 patients, including 9 at stage C and 11 at stage D per the GOLD classification, with histories of smoking were recruited and received cell-based therapy. The patients who received UC-MSC treatment showed significant reductions in Modified Medical Research Council scores, COPD assessment test scores, and the number of pulmonary exacerbations 6 months post administration. The results of the second trial using UC-MSCs showed that the mean FEV_1_/FVC ratios were increased along with improvements in SGRQ scores and 6MWTDs at three months post administration.^230^ Although thorough assessments of the effectiveness of UC-MSCs are still in the early stages, the number of trials using UC-MSCs for the treatment of COPD is increasing steadily, with larger sample sizes and stronger designs (randomized or matched case–control studies), providing a data set strongly supporting the future applications of UC-MSCs.^231^

The ongoing pandemic of the 21st century, the COVID-19 pandemic, emerged as a major pulmonary health problem worldwide, with a relatively high mortality rate. Numerous studies, reviews, and systematic analyses have been conducted to discuss and expand our knowledge of the virus and propose different mechanisms by which the virus could alter the immune system.^232^ One of the most critical mechanisms is the generation of cytokine storms, which result from the initiation of hyperreactions of the adaptive immune response to viral infection.^233^ These cytokine storms are formed by the establishment of waves of hypercytokinaemia generated from overreactive immune cells, which enhance their expression of TNF-α, IL-6, and IL-10, preventing T-lymphocyte recruitment and proliferation and culminating in T-lymphocyte apoptosis and T-cell exhaustion. In COVID-19, once a cytokine storm is formed, it spreads from an initial focal area through the body via circulation, which has been discussed in a comprehensive review by Jamilloux et al.^234^ At the time of writing this review, there were 74 clinical trials using MSCs from UC (29 trials; including WJ-derived MSCs (WJ-MSCs) and placenta-derived MSCs (PL-MSCs)), AT (15 trials), and BM (11 trials) (comprehensive review^171,235^). Hence, UC-MSCs have emerged as the most common MSCs for the treatment of COVID-19, with a total of 1047 patients participating in these trials. Among these trials, 15 completed trials using UC-MSCs (including WJ- and PL-MSCs) have been reported, with clinical data from approximately 600 recruited patients.^232^ Eight of these 15 studies used allogenic UC-MSC transplantation to treat critically ill patients.^236^ A list of case reports using UC-MSCs showed that the treatments were safe and well-tolerated in 14 patients with COVID-19, with the primary outcomes including increased percentages and numbers of T cells,^237,238^ improved respiratory and renal functions,^239^ reductions in inflammatory biomarker levels,^240^ and positive outcomes in the PaO_2_/FiO_2_ ratio.^240^ In a pilot study conducted in ten patients with severe COVID-19, a single dose of UC-MSCs was safe and improved clinical outcomes, although the study did not investigate whether multiple doses of UC-MSCs could further improve the outcomes.^241^ Two trials without a control group were conducted in 47 patients, and the results indicated that UC-MSCs were safe and feasible for the treatment of patients with COVID-19.^235,242^ A single-center, open-label, individually randomized, standard treatment-controlled trial was performed in 41 patients (12 patients assigned to the UC-MSC group), and the results showed that significant improvements in C-reactive protein levels, IL-6 levels, oxygen indices, and lymphocyte numbers were found in the MSC groups. Chest computed tomography (CT) illustrated significant reductions in lung inflammatory responses as reflected by CT findings, the number of lobes involved, and pulmonary consolidation.^238^ In a phase I trial conducted in 18 hospitalized patients with COVID-19, UC-MSCs were administered via an IV route in nine patients (five patients with moderate COVID-19 and 4 patients with severe COVID-19) at days 0, 3, and 6, with no treatment-related adverse events or severe adverse events.^243^ Only one patient in the UC-MSC group required mechanical ventilation, compared to four patients in the control group. However, the clinical outcomes, such as COVID-19 symptoms, laboratory test results, CT findings of lung damage, and pulmonary function test parameters, were improved in both groups. Interestingly, a 1-year follow-up of the same sample revealed that the patients who received UC-MSC administration improved in terms of whole-lung lesion volume compared to the control group.^244^ Moreover, chest CT at 12 months showed significant regeneration of lung tissue in the MSC-administered groups, whereas lung fibrosis was found in all patients in the control group. This finding is of interest because it indicates that a long time is needed to detect the regenerative functions of MSC-based therapy, as the biological process to enhance lung tissue regeneration occurs relatively slowly and requires multiple steps. The effects of UC-MSCs in the attenuation and prevention of the development of cytokine storms were illustrated in an interventional, prospective, three-parallel arm study with two control arms conducted in 30 patients in moderate and critical clinical conditions.^245^ The results indicated a significant decrease in proinflammatory cytokines (IFNγ, IL-6, IL-17A, IL-2, and IL-12) and an increase in anti-inflammatory cytokines (IL-10, IL-13, and IL-1ra), suggesting that UC-MSCs might participate in the prevention of cytokine storm development. Lanzoni et al. performed a double-blind, randomized, controlled trial and found that UC-MSC infusions significantly decreased cytokine levels at day 6 and improved survival in patients with COVID-19 with ARDS. In this trial, 24 patients were randomized and assigned 1:1 to receive either MSCs or placebo.^246^ MSC treatment was associated with a significant improvement in the survival rate without serious adverse events. To date, other trials conducted using UC-MSCs as the main MSCs provide a solid data set on their safety and efficacy in preventing the development of cytokine storms, reducing the inflammatory response, improving pulmonary function, reducing intensive care unit (ICU) stay duration, enhancing lung tissue regeneration, and reducing lung fibrosis progression.^240,247–249^ In two large cohort studies (phase I with 210 patients and phase II with 100 patients), the volume of lung lesions and solid component injuries of patients’ lungs were reduced significantly after the administration of UC-MSCs,^250^ and clinical symptoms and inflammatory levels were improved.^251^ Of the 26 reported clinical trials for the treatment of COVID-19 with MSCs, 1 study used AT-MSCs as the main MSCs.^236^ Thirteen COVID-19 adult patients under invasive mechanical ventilation who had received previous antiviral and/or anti-inflammatory treatments (including steroids, lopinavir/ritonavir, hydroxychloroquine, and/or tocilizumab, among others) were treated with allogeneic AT-MSCs. With a mean follow-up time of 16 days after infusion, 9/13 patients’ clinical symptoms improved, and 7/13 patients were intubated. A decrease in inflammatory cytokines and an increase in immunoregulatory cells were also observed in patients, especially in the group of patients with overall clinical improvement. Although there is a lack of clinical efficacy data supporting the use of AT-MSCs in the treatment of patients with COVID-19, AT-MSCs are still potential candidates for inhibiting COVID-19 due to their high secretory activity, strong immune-modulatory effects, and homing ability.^252–254^

For ARDS, in a phase IIa trial, 60 patients with moderate to severe disease were randomized into 2 groups. A group of 40 patients received a single infusion of BM-MSCs at a dose of 1 × 10^6^ cells/kg body weight, and another 20 patients received a placebo.^255^ After 6 and 24 h of infusion, the decrease in plasma inflammatory cytokine levels in the MSC group was significantly greater than that in the placebo group. For severe pulmonary hypertension (PH) associated with BPD (BPD-PH), in a small trial, two preterm infants born at 26–27 weeks of age were intravenously administered heterologous BM-MSCs at a dose of 5 × 10^6^ cells per kg of body weight; the treatment reduced oxygen requirements and supported respiration in the infants.^256^ The administration of allogeneic AT-MSCs in the treatment of ARDS appeared to be safe and well-tolerated in 12 adult patients, but clinical outcomes were not observed.^257^ The results of two patients who received BM-MSCs showed that both patients had improved respiratory function and hemodynamic function and a reduction in multiorgan failure.^258^ Although the safety of BM-MSCs was confirmed in a multicentre, open-label, dose-escalation, phase I clinical trial (The Stem cells for ARDS treatment—START trial),^259^ no significant improvements were found in a phase II trial, including in respiratory function and ARDS conditions.^260^ The safety profile of UC-MSCs is also supported by the findings of a previous phase I clinical trial conducted in 9 patients, which showed that a single IV administration of UC-MSCs was safe and led to positive outcomes in terms of respiratory function and a reduction in the inflammatory response.^261^ The findings of this study were also supported by those of the REALIST (Repair of Acute Respiratory Distress with Stromal Cell Administration) trial, which further confirmed the maximum tolerated dose of allogeneic UC-MSCs in patients with moderate to severe ARDS.^262^

Although AT- and BM-MSCs have demonstrated therapeutic potential with similar mechanisms of action, UC-MSCs have emerged as potential candidates in the treatment of pulmonary diseases due to their ease of production as “off-the-shelf” products, rapid proliferation, noninvasive isolation methods, and supreme immunological regulation as well as anti-inflammatory effects.^263^ However, it is important to note that there is a need to conduct phase III clinical trials with larger cohorts and trials with at least two sources of MSCs in the treatment of pulmonary conditions to further confirm this speculation.^264^ Table 2 summarizes several clinical trials with published results discussed in this review.

**Table 2:** The reported clinical trials using MSCs from AT, BM, and UC in the treatment of respiratory diseases

Year	Disease	MSC source	No. of MSC-treated patients	Efficacy
2015^258^	ARDS	BM	2	- Failed to improve after both standard life support measures, including mechanical ventilation, and additional measures, including extracorporeal ventilation
2015^259^	ARDS	BM	9	- Safety evaluation without secondary outcomes
2019^260^	ARDS	BM	60	- Increase Acute Physiology and Chronic Health Evaluation III, minute ventilation, and PEEP
				- No difference in mortality between the treatment and control group.
2020^261^	ARDS	UC	9	- Reduction of inflammation
				- Increase in immune cells
2021^262^	ARDS	UC	9	- Reduction of inflammation
				- Increase clinical outcomes
2018^486^	BPD	BM	2	- No improvement after the treatment
2014^213^	BPD	UC	9	- Reduction in inflammation
				- Improvement in respiratory severity score
2020^215^	BPD	UC	4	- Reduction in fibrosis
				- All treated patients recovered
2021^216^	BPD	UC	12	- Reduction in inflammation
				- Improvement in secondary outcomes
2021^216^	BPD	UCB	33	- No significant improvement in the primary outcomes between the two groups.
				- Severe BPD patients were significantly improved with MSC transplantation.
2015^226^	COPD	AD	8	- Safety and feasibility
2017^225^	COPD	AD	12	- Safety
2018^224^	COPD	BM	9	- Fail to migrate to areas of emphysematous remodeling in the lung
				- Reduction of systemic immunological response
2011^218^	COPD	BM	4	- Improvement in Psychological condition and quality of life, and clinical condition
2013^219^	COPD	BM	4	- Improvement in FVC, 6MWD, and DLCO
2013^220^	COPD	BM	30	- Improvement in FVC, FEV1, 6MWD, and – Reduction in inflammation
2017^221^	COPD	BM	5	- Improvement in FVC, TLC, 6MWD, and DLCO
2020^230^	COPD	UC	5	- Improvement in SGRQ symptom, activity, and impact scores
2020^263^	COPD	UC	20	- Improvement in FEV1, FCV, 6MWT, but not significant
				- Improvement in Modified Medical Research Council and COPD assessment test in all patients
2021^175^	COVID-19	UC	65	- Decrease in lung lesion proportion
2020^235^	COVID-19	UC	16	- Improvement in oxygenation index
				- Increase in the number of lymphocytes
				- Reduction in inflammation
2020^238^	COVID-19	UC	12	- Clinical improvement
				- Increase the number of lymphocytes
				- Decrease in inflammatory cytokines and CRP
				- Improvement in CT score
2020^239^	COVID-19	UC	1	- Improvement in renal function
				- Increase in the number of lymphocytes
2020^243^	COVID-19	UC	9	- Improvement in PaO_2_/FiO_2_ and CT score
				- Decrease inflammatory cytokine levels
2020^248^	COVID-19	UC	6	- Reduction in dyspnea, serum inflammatory cytokines
				- Increase in Sp02
				- Improvement in CT score
2021^242^	COVID-19	UC	31	- Reduction in inflammation
				- Improvement in CRP and CT score
2021^247^	COVID-19	UC	12	- Increase patients ‘survival
				- Reduction in inflammatory cytokines
2021^249^	COVID-19	UC	29	- Maintenance of SARS-CoV-2-specific antibodies and immune homeostasis
				- Reduction in CRP, proinflammatory cytokines, neutrophil extracellular traps
2021^250^	COVID-19	UC	65	- Reduction in lung lesion
				- Increase 6MWD
				- Improve CT score
2021^251^	COVID-19	UC	210	- Increase high survival
				- Improvement in SaO2
2020^237^	COVID-19	WJ	1	- Decrease in inflammatory cytokines and CRP
				- Increase in number of CD3+, CD4+, CD8+
2021^240^	COVID-19	WJ	5	- Reduction in inflammation
				- Improvement in CRP and CT score
2021^245^	COVID-19	WJ	10	- Improvement in CRP
				- Decrease in inflammation
2019^487^	Idiopathic pulmonary fibrosis	BM	10	- Improvement in FVC, 6MWD, and DLCO
2016^222^	LVES	BM	7	- Improvement in FEV1
				- Increase in number of CD3+ in alveolar septa and CD31+ in the alveolar septum,

*6MWD* 6-min walk distance, *ARDS* acute respiratory distress syndrome, *BPD* bronchopulmonary dysplasia, *COPD* chronic obstructive pulmonary dysplasia, *CRP* C-reactive protein, *CT* computed tomography scan, *DLCO* diffusing capacity for CO, *FCV* flow-controlled ventilation, *FEV*_*1*_ forced expiratory volume in 1 s, *FiO*_*2*_ fraction of inspired oxygen, *FVC* forced vital capacity, *PaO*_*2*_ partial pressure of oxygen, *PEEP* positive end-expiratory pressure, *SGRQ* St George’s Respiratory Questionnaire, *TLC* total lung capacity

## Endocrine disorders, infertility/reproductive function recovery, and skin burns: should we consider AT-MSCs as the main MSCs based on their origin?

### Endocrine disorders

The human body maintains function and homeostatic regulation via a complex network of endocrine glands that synthesize and release a wide range of hormones. The endocrine system regulates body functions, including heartbeat, bone regeneration, sexual function, and metabolic activity. Endocrine system dysregulation plays a vital role in the development of diabetes, thyroid disease, growth disorder, sexual dysfunction, reproductive malfunction, and other metabolic disorders. The central dogma of regenerative medicine is the use of adult stem cells as a footprint for tissue regeneration and organ renewal. The functions of these stem cells are tightly regulated by microenvironmental stimuli from the nervous system (rapid response) and endocrine signals via hormones, growth factors, and cytokines. This harmonized and orchestrated system creates a symphony of signals that directly regulate tissue homeostasis and repair after injury. The disruption of these complex networks results in an imbalance of tissue homeostasis and regeneration that can lead to the development of endocrine disorders in humans, such as diabetes, sexual hormone deficiency, premature ovarian failure (POF), and Asherman syndrome.

In recent years, obesity and diabetes (type 1 diabetes mellitus (T1DM) and type 2 diabetes mellitus (T2DM)) have been the two biggest challenges in endocrinology research, and the application of MSCs has emerged as a novel approach for therapeutic consideration. T1DM is characterized by the autoimmune destruction of pancreatic β-cells, whereas T2DM is defined as a combination of insulin resistance and pancreatic insulin-producing cell dysfunction. Regenerative medicine seeks to provide an exogenous cell source for replacing damaged or lost β-cells to achieve the goal of stabilizing patients’ blood glucose levels. To date, there are 28 clinical trials using MSCs in the treatment of T1DM (http://www.clinicaltrials.gov, searched in October 2021), among which three trials were completed using autologous BM-MSCs (NCT01068951), allogeneic BM-MSCs (NCT00690066), and allogeneic AT-MSCs (NCT03920397). Interestingly, UC-MSCs were the most favored MSCs for the remaining trials. All published studies confirmed the safety of MSC therapy in the treatment of T1DM with no adverse events. The first study using autologous BM-MSCs showed that patients who were randomized into the MSC-administration group showed an increase in C-peptide levels in response to a mixed-meal tolerance test (MMTT) in comparison to the control group.^265^ Unfortunately, there was no significant improvement in C-peptide levels, HbA1_C_ or insulin requirements. The use of autologous AT-MSCs in combination with vitamin D was safe and improved HbA1_C_ levels 6 months post administration.^266^ WJ-MSCs were used as the main MSCs for the treatment of new-onset T1DM, which showed a significant improvement in both HbA1_C_ and C-peptide levels when compared to those of the control group at three and six months post administration.^267,268^ The combination of allogeneic WJ-MSCs with autologous BM-derived mononuclear cells improved insulin secretion and reduced insulin requirements in patients with T1DM.^269^ In terms of T2DM, 23 studies were registered on clinicaltrials.gov (searched in October 2021), with six completed studies (three studies used BM-MSCs and three studies used allogeneic UC-MSCs). Although the number of studies using MSCs for the treatment of T2DM is small, their findings support the safety of MSCs, with no severe adverse events observed during the course of these studies.^270^ It was confirmed that MSC therapy potentially reduced fasting blood glucose and HbA1_C_ levels and increased C-peptide levels. However, these effects were short-term, and multiple doses were required to maintain the MSC effects. Interestingly, the autologous MSC approach in the treatment of patients with diabetes in general is hampered, as both BM-MSCs and AT-MSCs isolated from patients with diabetes showed reduced stemness and functional characteristics.^271,272^ In addition, the durations of diabetes and obesity are strongly associated with autologous BM-MSC metabolic function, especially mitochondrial respiration, and the accumulation of mitochondrial DNA, which directly interfere with the functions of BM-MSCs and reduce the effectiveness of the therapy.^271^ Therefore, the allogeneic approach using MSCs from healthy donors provides an alternative approach for stem cell therapy in the treatment of patients with diabetes.

### Infertility and reproductive function recovery

Modern society is increasingly facing the problem of infertility, which is defined as the inability to become pregnant after more than 1 year of unprotected intercourse.^273^ This problem has emerged as an important worldwide health issue and social burden. Assisted reproductive techniques and in vitro fertilization technology have recently become the most effective methods for the treatment of infertility in humans, but the use of these approaches is limited, as they cannot be applied in patients with no sperm or those who are unable to support implantation during pregnancy, they are associated with complications, they are time-consuming and expensive, and they are associated with ethical issues in certain territories.^274^ Numerous conditions are related to infertility, including POF, nonobstructive azoospermia, endometrial dysfunction, and Asherman syndrome. Recent progress has been illustrated in preclinical studies for the potential applications of stem cell-based therapy for reproductive function recovery, especially recent studies in the field of MSCs, which provide new hope for patients with infertility and reproductive disorders.^275^

POF is characterized by a loss of ovarian activity during middle age (before 40 years old) and affects 1–2% of women of reproductive age.^276^ Patients diagnosed with POF exhibit oligo-/amenorrhea for at least 4 months, with increased levels of follicle-stimulating hormone (FSH) (>25 IU/L) on two occasions more than 1 month apart.^277^ Diverse factors, such as genetic backgrounds, autoimmune disorders, environmental conditions, and iatrogenic and idiopathic situations, have been reported to be the cause of POF.^278^ POF can be treated with limited effectiveness via psychosocial support, hormone replacement intervention, and fertility management.^279^ MSCs from AT, BM, and UC have been used in the treatment of POF, with improvements in ovarian function in preclinical studies using chemotherapy-induced POF animal models. The early published POF study using BM-MSCs as the main cell source is a single case report in which a perimenopausal woman showed an improvement in follicular regeneration, and increased AMH levels resulted in a successful pregnancy followed by delivery of a healthy infant.^280^ A report using autologous BM-MSCs in two women with POF illustrated an increase in baseline estrogen levels and the volume of the treated ovaries along with amelioration of menopausal symptoms.^281^ The clinical procedures used in this early trial were invasive, as patients underwent two operations: (1) BM aspiration and (2) laparoscopy. A similar approach was used in two trials conducted in 10 women with POF (age range from 26–33 years old) and 30 patients (age from 18 to 40 years old).^282^ A later study investigated two different routes of cell delivery, including laparoscopy and the ovarian artery, but the results have not been reported at this time.^282^ Based on the positive outcomes of the mouse model, an autologous stem cell ovarian transplantation (ASCOT) trial was deployed using BM-derived stem cells with encouraging observations of improved ovarian function, as determined by elevated levels of AMH and AFC in 81.3% of participants, six pregnancies, and the successful delivery of three healthy babies.^283^ A randomized trial (NCT03535480) was conducted in 20 patients with POF aged less than 39 years to further elaborate on the results of the ASCOT trial.^284^ To date, there are no completed trials using AT-MSCs or UC-MSCs in the treatment of patients with POF, limiting the evaluation of these MSCs in the treatment of POF. The speculated reason is that POF is a rare disease, affecting 1% of women younger than 40 years, and with improvements in assisted productive technology, patients have several alternative options to enhance the recovery of reproductive function.^285^

### Wound healing and skin burns

Burns are the fourth most common injury worldwide, affecting ~11 million people, and are a major cause of death (180,000 patients annually). The severity of burns is defined based on the percentage of surface area burned, burn depth, burn location and patient age, and burns are usually classified into first-, second-, third-, and fourth-degree burns on the basis of their severity.^286^ Postburn recovery depends on the severity of the burn and the effectiveness of treatment. Rapid healing may occur over weeks, while alternatively, healing can take months, with the ultimate result being scar formation and disability in patients with severe burns. Different from mechanical injury, burn injury is an invasive progression of damage to tissue at the burn site, including both mechanical damage to the skin surface and biological damage caused by natural apoptosis that prolongs excessive inflammation, oxidative stress, and impaired tissue perfusion.^287^ To date, completely reversing the devastating damage of severe burns remains unachievable in medicine, and stem cell therapy provides an alternative option for patients with burn injury. The first case report of the use of BM-MSCs to treat a 45-year-old patient with burns on 40% of their body demonstrated the safety of the therapy and showed partial improvements in vascularization at the wound site and reduced coarse cicatrices.^288,289^ Later, patients with second- and third-degree burns as well as deep burns were treated using either autologous BM-MSCs or allogeneic BM-MSCs by spraying the MSCs onto the burn sites or adding MSCs over a dermal matrix sheet to cover the wound. The results in these case reports revealed the potential efficacy of MSC-based therapy, which not only enhanced the speed of wound recovery but also reduced pain and improved blood supply without introducing infection.^288,290,291^ In 2017, a study conducted in 60 patients with 10–25% of their total body surface areas burned treated with either autologous BM-MSCs or UC-MSCs showed that both MSC types improved the rate of healing and reduced the hospitalization period.^292^ The drawback of BM-MSCs in the treatment of burns is the invasive harvesting method, which causes pain and possible complications in patients. Hence, treatment with allogeneic MSCs obtained from healthy donors is the method of choice, and AT- and UC-MSCs are two suitable candidates for this option. To date, a limited number of clinical trials have been conducted using MSC therapy. These trials have several limitations in trial design, such as a lack of a negative control group and blinding, small sample sizes, and the use of standardized measurement tools for burn injury and wound healing. Currently, AT-MSCs are being used in seven ongoing phase I and II trials in the treatment of burns. Hence, it is important to note that among the most widely studied MSCs, AT-MSCs have advantages over BM-MSCs when obtained from an allogeneic source, while their abilities in burn treatment remain to be determined. The main MSCs that should be used in the regeneration of burn tissue remain undefined (Table 3), and we observed the trend that AT-MSCs are more suitable candidates due to their biological nature, which contributes to the generation of keratinocytes and secretion profiles that strongly enhance the skin regeneration process.^293–296^

**Table 3:** The reported clinical trials using MSCs from AT, BM, and UC in the treatment of the endocrinological disorder, reproductive disease, and skin healing

Year	Type of disease	Cell source	No. of treated patients	Efficacy
2014^265^	Type 1 diabetes	BM	9	- No significant improvement compared to control group in HbA1c, insulin doses per kilogram, fasting C-peptide
				- 3/9 MSC-treated patients decreased their peak C-peptide or AUC response to the MMTT while 8/9 patients decreased in peak C-peptide, and 7/9 decreased in AUC response in the control group
2021^266^	Type 1 diabetes	AD	7	Significant improvement compared to before transplantation in basal C-peptide and HbA1C
2013^268^	Type 1 diabetes	WJ	15	Significant improvement over the control group in HbA1c and fasting C-peptide
2015^269^	Type 1 diabetes	WJ + BM	21	The metabolic measures improved in treated patients:
				+ AUC C-Pep increased 105.7% (*P* = 0.00012);
				+ insulin area under the curve increased 49.3% (*P* = 0.01)
				+ HbA1c decreased 12.6% (*P* < 0.01)
				+ Fasting glycemia decreased 24.4% (*P* < 0.002)
				+ Daily insulin requirements decreased 29.2% (*P* = 0.001)
2021^271^	Type 2 diabetes	BM	25	A slight reduction in HbA1c levels was observed in the first 3 months after administration, but the level returned to normal after 6 months and even increased
2005^288^	Skin burns	BM	1	The improvement in vascularization at the wound site and reduced coarse cicatrices
2012^290^	Skin burns	BM	1	The areas treated with autologous BM-MSCs combined with transplantation of split skin were less likely to have contraction of the skin grafts.
2008^291^	Skin wounds	BM	20	The wound mostly healed in 18 of the 20 patients showed the BM-MSCs transplantation effectively
2017^292^	Skin burns	BM-MSC & UC-MSC	40	The significantly improved rate of healing in both BM-MSC and UC-MSC groups as compared to traditionally treated group in percent of burn extent (%), hospitalization time.
2018^280^	Premature ovarian insufficiency	BM-MSC	1	- The AMH level improved from 0.4 to 0.9 ng/mL
				- The improvement of follicular regeneration resulted in a successful pregnancy followed by the delivery of a healthy infant
2020^281^	Premature ovarian failure	BM-MSC	2	The increase in baseline estrogen levels and amelioration of menopausal symptoms
2018^422^	Premature ovarian insufficiency	UC-MSC	14	The elevated estradiol concentrations, improved follicular development, and increased number of antral follicles
2016^488^	Premature ovarian insufficiency	BM-MSC	10	The improvement in Edessy ovarian reserve score (EORS) and increased pregnancy capacity
2016^489^	Premature ovarian insufficiency	BM-MSC	30	86.7% of patients showed a fall in FSH levels and a rise in estrogen and AMH levels after 4 weeks of injection
2018^283^	Premature ovarian insufficiency	BM-MSC	15	- The significant improvement in AFC and AMH after treatment.
				- Increased the number of stimulable antral follicles and oocytes
				- Ovarian function improved in 81.3% of women

*AFC* antral follicle count, *AMH* anti-Müllerian hormone, *AUC* area under the curve (oral glucose tolerance test), *FSH* follicle-stimulating hormone, *HbA1C* hemoglobin A1C, *MMTT* mixed-meal tolerance test

## MSC applications in cardiovascular disease: a promising but still controversial field

In the last two decades, great advancements have been achieved in the development of novel regenerative medicine and cardiovascular research, especially stem cell technology.^297^ The discovery of human embryonic stem cells and human induced pluripotent stem cells (hiPSCs) opened a new door for basic research and therapeutic investigation of the use of these cells to treat different diseases.^298^ However, the clinical path of hiPSCs and hiPSC-derived cardiomyocytes in the treatment of cardiovascular diseases is limited due to the potential for teratoma formation with hiPSCs and the immaturity of hiPSC-derived cardiomyocytes, which might pose a risk of cancer formation,^299^ arrhythmia, and cardiac arrest to patients.^300^ A recently emerged stem cell type is adult stem cells/progenitor cells, including MSCs, which can stimulate myocardial repair post administration due to their paracrine effects. Promising results of MSC-based therapy obtained from preclinical studies of cardiac diseases enhance the knowledge and strengthen the clinical research to investigate the safety and efficacy in a clinical trial setting. There are papers that discuss the importance of MSC therapy in the treatment of cardiovascular diseases, with the following references being highly recommended.^301–306^ To date, 36 trials have evaluated the therapeutic potential of MSCs in different pathological conditions, with the most prevalent types being BM-MSCs (25 trials), followed by UC-MSCs (7 trials) and AT-MSCs (4 trials).^303^ However, the reported results are contradictory and create controversy about the efficacy of the treatments.

One of the first trials using MSCs in the treatment of chronic heart failure was the Cardiopoietic Stem Cell Therapy in Heart Failure (C-CURE) trial, a multicentre, randomized clinical trial that recruited 47 patients. The trial findings supported the safety of BM-MSC therapy and provided a data set that demonstrated improvements in cardiovascular scores along with New York Heart Association functional class, quality of life, and general physical health.^307^ Despite these encouraging results in the phase I trial, the treatment failed to achieve the primary outcomes in the phase II/III trial (CHART-1 trial), including no significant improvements in cardiac structure or function or patient quality of life.^308^ A positive outcome was also found in a phase I/II, randomized pilot study called the POSEIDON trial, which was the first trial to demonstrate the superior effectiveness of the administration of allogeneic BM-MSCs compared to allogeneic MSCs from other sources.^309,310^ Published results from the MSC-HF study, with 4 years of follow-up results,^311,312^ and the TRIDENT study^313^ illustrated the positive outcomes of BM-MSCs in the treatment of heart failure. However, a contradictory result from the recently published CONCERT-HF trial demonstrated that the administration of autologous BM-MSCs to patients diagnosed with chronic ischemic heart failure did not improve left ventricular function or reduce scar size at 12 months post administration, but the patient’s quality of life was improved.^314^ This observation is similar to that of the TAC-HFT trial^315^ but completely different from the reported results of the MSC-HF trial. A comprehensive investigation is still needed to determine the reasons behind these contradictory results. The largest clinical trial to date using BM-MSCs is the DREAM-HF study, which was a randomized, double-blind, placebo-controlled, phase III trial that was conducted at 55 sites across North America and recruited a total of 565 patients with ischemic and nonischaemic heart failure.^172^ Although recent reports from the sponsor confirmed that the trial missed its primary endpoint (a reduction in recurrent heart failure-related hospitalization), other prespecified endpoints were met, such as a reduction in overall major adverse cardiac events (including death, myocardial infarction, and stroke).^306^ Thus, a complete report from the DREAM-HF trial will provide pivotal data supporting the therapeutic potential of BM-MSCs in the treatment of heart failure and open a new path for the FDA to approve cell-based therapy for cardiovascular diseases.

The early trial using AT-derived cells was the PRECISE trial, which was a phase I, randomized, placebo-controlled, double-blind study that examined the safety and efficacy of adipose-derived regenerative cells (ADRCs) in the treatment of chronic ischemic cardiomyopathy.^316^ ADRCs are a homogenous population of cells obtained from the vascular stromal fraction of AT, which contains a small proportion of AT-MSCs.^317^ Although the study supported the safety of ADRC administration and illustrated a preserved functional capacity (peak VO_2_) in the treated group and improvements in heart wall motion, neither poor left ventricle (LV) volume nor poor left ventricular ejection fraction (LVEF) was ameliorated. The follow-up trial of the PRECISE trial, called the ATHENA trial, was conducted in 31 patients, although the study was terminated prematurely because two cerebrovascular events occurred, which were not related to the cell product itself.^318^ The results of the study illustrated increases in functional capacity, hospitalization rate, and MLHFQ scores, but the LV volume and LVEF were not significantly different between the two groups. Kastrup and colleagues conducted the first in vitro expanded AT-MSC trial in ten patients with ischemic heart disease and ischemic heart failure in 2017. The results confirmed that ready-to-use AT-MSCs were well-tolerated and potentially effective in the treatment of ischemic heart disease and heart failure.^319^ Comparable results of AT-MSCs were also reported from the MyStromalCell Trial, which was a randomized placebo-controlled study. In this trial, 61 patients were randomized at a 2:1 ratio into two groups, with the results showing no significant difference in the primary endpoint, which was a change in the maximal bicycle exercise tolerance test (ETT) score from baseline to 6 months post administration.^320^ A 3-year follow-up report from the MyStromalCell Trial confirmed that patients who received AT-MSC administration maintained their preserved exercise capacity and their cardiac symptoms improved, whereas the control group experienced a significant reduction in exercise performance and a worsened cardiovascular condition.^321^

UC-MSCs are potential allogeneic cells for the treatment of cardiovascular disease, as they are “ready to use” and easy to isolate, they rapidly proliferate, and they secrete hepatocyte growth factors,^322^ which are involved in cardioprotection and cardiovascular regeneration.^323^ The pilot study using UC-MSCs in 30 patients with heart failure, called the RIMECARD trial, was the first reported trial for which the results supported the effectiveness of UC-MSCs, as seen in the improved ejection fraction, left ventricular function, functional status, and quality of life in patients administered UC-MSCs.^324^ Encouraging results reported from a phase I/II HUC-HEART trial^325^ showed improvements in LVEF and reductions in the size of the injured area of the myocardium. However, the opposite observations were also reported from a recently published phase I randomized trial using a combination of UC-MSCs and a collagen scaffold in patients with ischemic heart conditions, in which the size of fibrotic scar tissue was not significantly reduced.^326^

Although MSCs from AT, BM, and UC have proven to be safe and feasible in the treatment of cardiovascular diseases, the correlation between the MSC types and their therapeutic potentials is still uncertain because different results have been reported from different clinical trials (Table 4). The mechanisms by which MSCs participate in recovery and enhance myocardial regeneration have been discussed comprehensively in a recently published review;^305,327^ therefore, they will not be discussed in this review. In fact, the challenges of MSC-based therapy in cardiovascular diseases have been clearly described previously,^328^ including (1) the lack of an in vitro evaluation of the transdifferentiation potential of MSCs to functional cardiac and endothelial cells,^329^ (2) the uncontrollable differentiation of MSCs to undesirable cell types post administration,^330^ and (3) the undistinguishable nature of MSCs derived from different sources with various levels of differentiation potential.^331^ Therefore, the applications of MSC-based therapy in cardiovascular disease are still in their immature stage, with potential benefits to patients. Thus, there is a need to conduct large-scale, well-designed randomized clinical trials not only to confirm the therapeutic potential of MSCs from various sources but also to enhance our knowledge of cardiovascular regeneration post administration.

**Table 4:** The reported clinical trials using MSCs from AT, BM, and UC in the treatment of cardiovascular diseases

Year	Disease	MSC source	No. of treated patients	Efficacy
2013^307^	Heart failure	BM	21	- Improvement in LVEF and MLHFQ
				- Decrease in LVESV and LVEDV
				- Increase in 6MWT
				- No evidence of increased cardiac or systemic toxicity
2017^308^	Ischemic heart failure	BM	120	- Improvement in LVEF, ESV, LEVDV, and MLHFQ
2012^309^	ischemic cardiomyopathy	BM	30	- Functional improvement (change in 6MWT, MLHFQ, and NYHA classification)
2017^310^	Nonischemic Dilated Cardiomyopathy	BM	34	- Increase in 6MWT, ejection fraction
				- Improvement in MLHFQ
				- Reduction in inflammatory cytokine, TNF-a
2015^311^	Severe ischemic heart failure	BM	60	- Significant Improvement in LVEF, ESV, stroke volume, and myocardial mass. No difference in NYHA class, 6MWT, and Kansas City cardiomyopathy questionnaire after 6-month follow-up
2020^312^	Severe ischemic heart failure	BM	60	- Significant improvements in LVEF, LVESV, stroke volume and myocardial mass. Significant reduction in the amount of scar tissue and quality of life score after 12 months’ of follow-up.
				- Significantly fewer hospitalizations for angina after 4 years of follow-up
2017^313^	Ischemic Cardiomyopathy	BM	30	- Reduction in scar size
				- Increase in ejection fraction when using 100 million dose
2021^314^	Ischemic heart failure	BM	25	- Improvement in clinical outcomes, including MACE and quality of life
				- No improvement in LVEF, left ventricular, scar size, 6MWT, and oxy peak oxygen consumption did not differ between groups
2013^315^	ischemic cardiomyopathy	BM	19	- Improvement in MLHF, 6MWT, regional myocardial function
				- No change in ejection fraction and left ventricular chamber volume
2014^316^	Chronic ischemic cardiomyopathy	AD	21	- No significant change in LVEF, SPECT, and rest total severity score
				- Increase in LV total mass
				- Improvement in WMSI
				- Preserved MVO_2_ and METs
2017^318^	Chronic myocardial ischemia	AD	15	- Significant improvement in LVEF, ESV, LEVDV, and MLHFQ
2017^319^	Ischemic heart failure	AD	10	- Improvement in LVEF, LVSEV, 6MWT, NYHA class
				- No difference in KKCQ scores and CCS class
2017^320^	Chronic Ischemic Heart Disease	AD	41	- Improvement in Exercise capacity compared to placebo but not significant
2019^321^	Refractory angina	AD	41	- Improvement in cardiac symptoms but no change in exercise capacity
2017^324^	Heart failure	UC	15	- Improvements in LVEF compared to baseline at 3 months’follow-up
				- No changes in left ventricular volumes
2020^325^	Chronic ischemic cardiomyopathy	UC	26	- Improvement in LVEF, 6MWT, and NYHA
2020^326^	Chronic ischemic heart disease	UC	32	- Improvement in LVEF, MLHF, and NYHA
				- Decrease infarct size

*6MWT* 6-min walk test, *CCS* Canadian Cardiovascular Society, *ESV* end-systolic volume, *LVEDV* left ventricular end-diastolic chamber volume, *LVEF* left ventricular ejection fraction, *LVESV* left ventricular end-systolic volume, *MACE* major adverse cardiovascular events, *MET* metabolic equivalents, *MLHF* Minnesota Living with Heart Failure, *MLHFQ* Minnesota Living with Heart Failure Questionnaire, *MVO*_*2*_ maximal oxygen consumption, *NYHA* New York Heart Association, *SPECT* single photon emission computed tomography, *TNF-α* tumor necrosis factor alpha, *WMSI* wall motion score index

## Proposed mechanism of BM-MSCs in the treatment of acquired brain and spinal injury

**Fig. 3:**
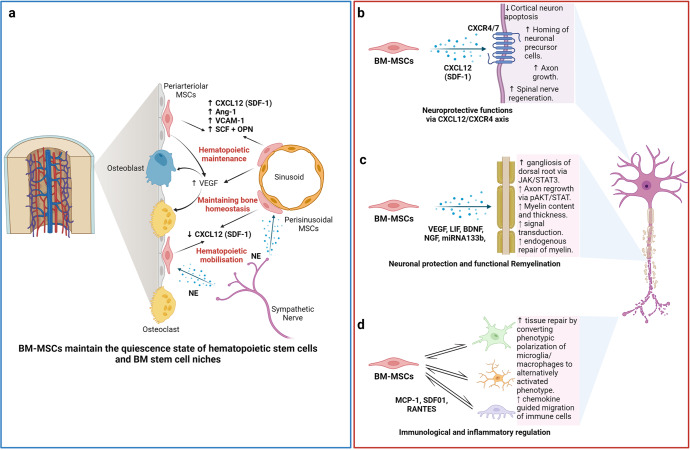
The nature of the “stem niche” of bone marrow-derived mesenchymal stem cells (BM-MSCs) supports their therapeutic potential in neuron-related diseases. **a** Bone marrow is a complex stem cell niche regulated directly by the central nervous system to maintain bone marrow homeostasis and haematopoietic stem cell (HSC) functions. MSCs in bone marrow respond to the environmental changes through the release of norepinephrine (NE) from the sympathetic nerves that regulate the synthesis of SDF-1 and the migration of HSCs through the sinusoids. The secretion of stem cell factors (SCFs), VCAM-1 and angiotensin-1 from MSCs also plays a significant role in the maintenance of HSCs. **b** BM-MSCs have the ability to produce and release SDF-1, which directly contributes to neuroprotective functions at the damaged site through interaction with its receptors CXCR4/7, located on the neuronal membrane. **c** Neuronal protection and the functional remyelination induced by BM-MSCs are also modulated by the release of a wide range of growth factors, including VEGF, BDNF, and NGF, by the BM-MSCs. **d** BM-MSCs also have the ability to regulate neuronal immune responses by direct interaction or paracrine communication with microglia. Figure was created with BioRender.com

Bones are complex structures constituting a part of the vertebrate skeleton, and they play a vital role in the production of blood cells from HSCs. Similar to the functions of most vertebrate organs, bone function is tightly regulated by its constituents and by long-range signaling from AT and the adrenal glands, parathyroid glands, and nervous system.^332^ The central nervous system (CNS) orchestrates the voluntary and involuntary input transmitted by a network of peripheral nerves, which act as the bridge between the nervous system and target organs. The CNS controls involuntary responses via the autonomic nervous system (ANS), consisting of the sympathetic nervous system and the parasympathetic nervous system, and voluntary responses via the somatic nervous system. The ANS penetrates deep into the BM cavity, reaching the regions of hematopoietic activity to deliver neurotransmitters that tightly regulate BM stem cell niches.^333^ The BM microenvironment consists of various cell types that participate in the maintenance of HSC niches, which are composed of specialized cells, including BM-MSCs (Fig. 3a). The release of a specific neurotransmitter, circadian norepinephrine, from the sympathetic nervous system at nerve terminals leads to a reduction in the circadian expression of C–X-C chemokine ligand 12 (CXCL12, which is also known as stromal cell-derived factor-1 (SDF-1)) by Nestin^+^/NG2^2+^ BM-MSCs, resulting in the secretion of HSCs into the peripheral bloodstream.^334,335^ In fact, BM-MSCs play a significant role in the regulation of HSC quiescence and are closely associated with arterioles and sympathetic nervous system nerve fibers. Nestin-expressing BM-MSCs have been shown to express high levels of SDF-1, stem cell factor (SCF), angiopoietin-1 (Ang-1), interleukin-7, vascular cell adhesion molecule 1 (VCAM-1), and osteopontin (OPN), which are directly involved in the regulation and maintenance of HSC quiescence.^336^ The depletion of BM-MSCs in BM leads to the mobilization of HSCs into the peripheral bloodstream and spleen. The findings from a previous study demonstrated that reduced SDF-1 expression in norepinephrine-treated BM-MSCs resulted in the mobilization of CXCR4^+^ HSCs into circulation.^337^ The ability of BM-MSCs to produce SDF-1 is tightly related to their neuronal protective functions.^338^ SDF-1 is a member of a chemokine subfamily that orchestrates an enormous diversity of pathways and functions in the CNS, such as neuronal survival and proliferation. The chemokine has two receptors, CXCR4 and CXCR7, that are involved in the pathogenic development of neurodegenerative and neuroinflammatory diseases.^339^ In the damaged brain, SDF-1 functions as a stem cell homing signal, and in acquired immune deficiency syndrome (AIDS), SDF-1 has been reported to be involved in the protection of damaged neurons by preventing apoptosis. In a traumatic brain injury model, SDF-1 was found to function as an inhibitor of the caspase-3 pathway by upregulating the Bcl-2/Bax ratio, which in turn protects neurons from apoptosis.^340^ Moreover, the release of SDF-1 also facilitates cell recruitment, cell migration, and the homing of neuronal precursor cells in the adult CNS by activating the CXCR4 receptor.^341,342^ Existing data support that SDF-1 acts as the guiding signal for the regeneration of axon growth in damaged neurons and enhances spinal nerve regeneration.^343,344^ Hence, the ability of BM-MSCs to express SDF-1 in response to the neuronal environment provides a unique neuronal protective effect that could explain the potential therapeutic efficacy of BM-MSCs in the treatment of neurodegenerative diseases (Fig. 3b).

The migration of exogenous MSCs after systemic administration to the brain is limited by the physical blood–brain barrier (BBB), which is a selective barrier formed by CNS endothelial cells to restrict the passage of molecules and cells. The mechanism of molecular movement across the BBB is well established, but how stem cells can bypass the BBB and home to the brain remains unclear. Recent studies have reported that MSCs are able to migrate through endothelial cell sheets by paracellular or transcellular transport followed by migration to the injured or inflammatory site of the brain.^345,346^ During certain injuries or ischemic events, such as brain injury, stroke, or cerebral palsy, the integrity and efficiency of BBB protection is compromised, which allows MSC migration across the BBB via paracellular transport through the transient formation of interendothelial gaps.^347^ CD24 expression has been detected in human BM-MSCs, which are regulated by TGF-β3,^348^ allowing them to interact with activated endothelial cells via P-selectin and initiate the tethering and rolling steps of MSCs.^349^ Additionally, BM-MSCs express high levels of CXCR4 or CXCR7,^350,351^ which bind to integrin receptors, such as VLA-4, to activate the integrin-binding process and allow the cells to anchor to endothelial cells, followed by the migration of MSCs through the endothelial cell layer and basement membrane in a process called transmigration.^352^ This process is facilitated by the secretion of matrix metalloproteinases (MMPs), which degrade the endothelial basement membrane, allowing BM-MSCs to enter the brain environment.^353,354^ BM-MSCs can also regulate the integrity of the BBB via the secretion of tissue inhibitor of matrix metalloproteinase-3 (TIMP3), which has been shown to ameliorate the effects of a compromised BBB in traumatic brain injury.^355^ The secretion of TIMP3 from MSCs directly blocked vascular endothelial growth factor a (VEGF-a)-induced breakdown of endothelial cell adherent junctions, demonstrating the potential mechanism of BM-MSCs in the regulation of BBB integrity.

The therapeutic applications of BM-MSCs in neurodegenerative conditions have been significantly increased by the demonstration of BM-MSC involvement in axonal and functional remyelination processes. Remyelination is a spontaneous regenerative process occurring in the human CNS to protect oligodendrocytes, neurons, and myelin sheaths from neuronal degenerative diseases.^356^ Remyelination is considered a neuroprotective process that limits axonal degeneration by demyelination and neuronal damage. The first mechanism of action of BM-MSCs related to remyelination is the activation of the JAK/STAT3 pathway to regulate dorsal root ganglia development.^357^ It was reported that BM-MSCs secrete vascular endothelial growth factor-A (VEGF-A),^358^ brain-derived neurotrophic factor (BDNF), interleukin-6, and leukemia inhibitor factor (LIF), which directly function in neurogenesis and neurite growth.^357^ VEGF-A is a key regulator of hemangiogenesis during development and bone homeostasis. Postnatally, osteoblast- and MSC-derived VEGF plays a critical role in maintaining and regulating bone homeostasis by stimulating MSC differentiation into osteoblasts and suppressing their adipogenic differentiation.^359–361^ To balance osteoblast and adipogenic differentiation, VEGF forms a functional link with the nuclear envelope protein laminin A, which in turn directly regulates the osteoblast and adipocyte transcription factors Runx2 and PPARγ, respectively.^361,362^ In the brain, VEGF is a potent growth factor mediating angiogenesis, neural migration, and neuroprotection. VEGF-A, secreted from BM-MSCs under in vitro xeno- and serum-free culture conditions, is the most studied member of the VEGF family and is suggested to play a protective role against cognitive impairment, such as in the context of Alzheimer’s disease pathology or stroke.^363–365^ Recently, it was reported that the neurotrophic and neuroprotective function of VEGF is mediated through VEGFR2/Flk-1 receptors, which are expressed in the neuroproliferative zones and extend to astroglia and endothelial cells.^366^ In animal models of intracerebral hemorrhage and cerebral ischemia, the transfusion of Flk-1-positive BM-MSCs promotes behavioral recovery and anti-inflammatory and angiogenic effects.^367,368^ Moreover, supplementation with VEGF-A in neuronal disorders enhances intraneural angiogenesis, improves nerve regeneration, and promotes neurotrophic capacities, which in turn increase myelin thickness via the activation of the prosurvival transcription factor nuclear factor-kappa B (NF-kB). This activation, together with the downregulation of Mdm2 and increased expression of the pro-apoptotic transcription factor p53, is considered to be the neuroprotective process associated with an increased VEGF-A level.^369–371^ An analysis of microRNA (miRNA) in extracellular vesicles (EVs) secreted from BM-MSCs revealed that BM-MSCs release substantial amounts of miRNA133b, which suppresses the expression of connective tissue growth factor (CTGF) and protects hippocampal neurons from apoptosis and inflammatory injury^372–374^ (Fig. 3c).

In terms of immunoregulatory functions, the administration of human BM-MSCs into immunocompetent mice subjected to SCI or brain ischemia showed that BM-MSCs exhibited a short-term neuronal protective function against neurological damage (Fig. 3d). Further investigation demonstrated the ability of BM-MSCs to directly communicate with host microglia/macrophages and convert them from phenotypic polarization into alternative activated microglia/macrophages (AAMs), which are key players in axonal extension and the reconstruction of neuronal networks.^375^ Other studies have also illustrated that the administration of AAMs directly to the injured spinal cord induced axonal regrowth and functional improvement.^376^ The mechanism by which BM-MSCs activate the conversion of microglia/macrophages occurs through two representative macrophage-related chemokine axes, CCL2/CCR2 and CCL-5/CCR5, both of which exhibit acute or chronic elevation following brain injury or SCI.^377^ The CCL2/CCR2 axis contributed to the enhancement of inflammatory function, and BM-MSC-mediated induction of CCL2 did not alter the total granulocyte number (Fig. 3d). Although the chemokine-mediated mechanism of BM-MSCs in the activation of AAMs and enhanced axonal regeneration at the damage sites is evident, the direct mechanism by which the communication between BM-MSCs and the target cells results in these phenomena remains unclear, and further investigation is needed.

BM-MSCs also confer the ability to regulate the inflammatory regulation of the immune cells present in the brain by (1) promoting the polarization of macrophages toward the M2 type, (2) suppressing T-lymphocyte activities, (3) stimulating the proliferation and differentiation of regulatory T cells (Tregs), and (4) inhibiting the activation of natural killer (NK) cells. BM-MSCs secrete glial cell line-derived neurotrophic factor (GDNF), a specific growth factor that contributes directly to the transition of the microglial destructive M1 phenotype into the regenerative M2 phenotype during the neuroinflammatory process.^378^ A similar result was also found in AT-^379^ and UC-MSCs^380^ under neuroinflammation-associated conditions, suggesting that AT-, BM-, and UC-MSCs share the same mechanism in promoting macrophage polarization. In terms of T-lymphocyte suppression, compared to MSCs from AT and BM, UC-MSCs show the strongest potential to inhibit the proliferation of T-lymphocytes by promoting cell cycle arrest (G0/G1 phase) and apoptosis.^381^ In addition, UC-MSCs have been proven to be more effective in promoting the proliferation of Tregs^382^ and inhibiting NK activation.^383^ Although MSCs are well-known for their inflammatory regulatory ability, the mechanism is not exclusive to BM-MSCs, especially in neurological disorders.^384^

## Proposed mechanism of UC-MSCs in the treatment of pulmonary diseases and lung fibrosis

**Fig. 4:**
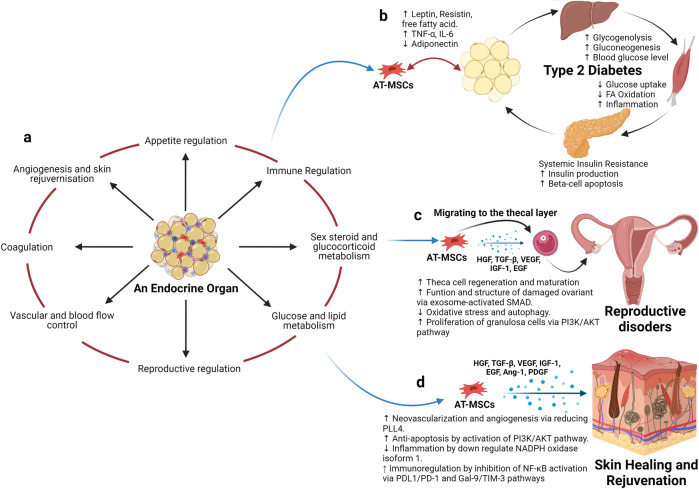
Adipose tissue-derived mesenchymal stem cells (AT-MSCs) and the nature of their tissue of origin support their use in therapeutic applications. **a** Adipose tissue is considered an endocrine organ, supporting and regulating various functions, including appetite regulation, immune regulation, sex hormone and glucocorticoid metabolism, energy production, the orchestration of reproduction, the control of vascularization, and blood flow, the regulation of coagulation, and angiogenesis and skin regeneration. **b** In terms of metabolic disorders, such as type 2 diabetes mellitus (T2DM), as adipose tissue is directly involved in the metabolism of glucose and lipids and the regulation of appetite, the detrimental effects of T2DM also alter the functions of AT-MSCs, which in turn, hampers their therapeutic effects. Hence, the use of autologous AT-MSCs is not recommended for the treatment of metabolic disorders, including T2DM, suggesting that allogeneic AT-MSCs from healthy donors could be a better alternative approach. **c** AT-MSCs are suitable for the treatment of reproductive disorders due to their unique ability to mobilize and home to the thecal layer of the injured ovary, enhance the regeneration and maturation of thecal cells, increase the structure and function of damaged ovaries via exosome-activated SMAD, decrease oxidative stress and autophagy, and increase the proliferation of granulosa cells via PI3K/AKT pathways. These functions are regulated specifically by growth hormones produced by AT-MSCs in response to the surrounding environment, including HGF, TGF-β, IGF-1, and EGF. **d** AT-MSCs are also good candidates for skin healing and regeneration as their growth factors strongly support neovascularization and angiogenesis by reducing PLL4, increase anti-apoptosis via the activation of PI3K/AKT pathways, regulate inflammation by downregulating NADPH oxidase isoform 1, and increase immunoregulation through the inhibition of NF-κB activation. The figure was created with BioRender.com

In contrast to AT-MSCs and BM-MSCs, UC-MSCs have lower expression of major histocompatibility complex I (MHC I) and no expression of MHC II, which prevents the complications of immune rejection.^385^ Moreover, as UC is considered a waste product after birth, with the option of noninvasive collection, UC-MSCs are easier to obtain and culture than AD- and BM-MSCs.^386^ These advantages of UC-MSCs have contributed to their use in the treatment of pulmonary diseases, especially during the rampant COVID-19 pandemic, as “off-the-shelf” products. Numerous pulmonary diseases have been the subject of applications of UC-MSCs, including BPD, COPD, ARDS, and COVID-19-induced ARDS. In BPD, premature infants are born before the alveolarization process, resulting in arrested lung development and alveolar maturation. Upon administration via an IV route, the majority of exogenous UC-MSCs reach the immature lung and directly interact with immune cells to exert their immunomodulatory properties via cell-to-cell interaction mechanisms (Fig. 4a). UC-MSCs interact with T cells via the PD-L1 ligand, which binds to the PD-1 inhibitory molecule on T cells, resulting in the suppression of CD3+ T-cell proliferation and effector T-cell responses.^387^ In addition, UC-MSCs also express CD54 (ICAM-1), which plays a crucial role in the immunomodulatory functions of T cells.^388^ Direct contact between UC-MSCs and macrophages via CD54 expression on UC-MSCs promotes the immune regulation of UC-MSCs via the regulation of phagocytosis by monocytes.^389^ Moreover, the contact of UC-MSCs with macrophages during proinflammatory responses increases the secretion of TSG-6 by UC-MSCs, which in turn promotes the inhibitory regulation of CD3+ T cells, macrophages, and monocytes by MSCs.^390^ Recently, upregulation of SDF-1 was described in neonatal lung injury, especially in layers of the respiratory epithelium.^391^ SDF-1 has been shown to participate in the migration and initiation of the homing process of MSCs via the CXCR4 receptors on their surface.^392^ It was reported that UC-MSCs express low levels of CXCR4, allowing them to induce SDF-1-associated migration processes via the Akt, ERK, and p38 signal transduction pathways.^393^ Hence, in BPD, the upregulation of SDF-1 together with the homing ability of UC-MSCs strongly supports the therapeutic effects of UC-MSCs in the treatment of BPD. Furthermore, UC-MSCs have the ability to communicate with immune cells via cell-to-cell contact to reduce proinflammatory responses and the production of proinflammatory cytokines (such as TGF-β, INF-γ, macrophage MIF, and TNF-α). The modulation of the human innate immune system by UC-MSCs is mediated by cell–cell interactions via CD54-LFA-1 that switch macrophage polarization processes, promoting the proliferation of M2 macrophages, which in turn reduce inflammatory responses in the immature lung.^394^ Moreover, UC-MSCs also have the ability to produce VEGF and hepatocyte growth factors (HGFs), promoting angiogenesis and enhancing lung maturation.^395^

COPD is characterized by an increase in hyperinflammatory reactions in the lung, compromising lung function and increasing the development of lung fibrosis. The mechanism by which UC-MSCs contribute to the response to COPD is inflammatory regulation (Fig. 4b). The administration of UC-MSCs prevented the infiltration of inflammatory cells in peribronchiolar, perivascular, and alveolar septa and switched macrophage polarization to M2.^396^ A significant reduction in proinflammatory cytokines, including IL-1β, TNF-α, and IL-8, was also observed following UC-MSC administration.^224^ MSCs, including UC-MSCs, have been reported to trigger the production of secretory leukocyte protease inhibitors in epithelial cells through the secretion of HGF and epidermal growth factor (EGF), which is believed to have beneficial effects on COPD.^397,398^ In addition to their inflammatory regulation ability, UC-MSCs exhibit antimicrobial effects through the inhibition of bacterial growth and the alleviation of antibiotic resistance during *Pseudomonas aeruginosa* infection.^399^ The combination of the regulation of the host immune response and the antimicrobial effects of UC-MSCs may be relevant for the prevention and treatment of COPD exacerbations, as inflammation and bacterial infections are important risk factors that significantly contribute to the morbidity and mortality of patients with COPD. In terms of regenerative functions, UC-MSCs were reported to be able to differentiate into type 2 alveolar epithelial cells in vitro and alleviate the development of pulmonary fibrosis via β-catenin-regulated cell apoptosis.^400^ Furthermore, UC-MSCs enhanced alveolar epithelial cell migration and proliferation by increasing matrix metalloproteinase-2 levels and reduced their endogenous inhibitors, tissue inhibitors of matrix metalloproteinases, providing a potential mechanism underlying their anti-pulmonary-fibrosis effects.^401,402^

In ARDS, especially that associated with COVID-19, the proinflammatory state is initiated by increases in plasma concentrations of proinflammatory cytokines, such as IL-1 beta, IL-7, IL-8, IL-9, IL-10, bFGF, granulocyte colony-stimulating factor (G-CSF), GM-CSF, IFN-γ, and TNF-α. The significant increases in the concentrations of these cytokines in patient plasma suggest the development of a cytokine storm, which is a leading cause of COVID-induced mortality. In addition to the immunomodulatory functions regulated via cell-to-cell interactions between UC-MSCs and immune cells, such as macrophages, monocytes, and T cells, UC-MSCs exert their functions via paracrine effects through the secretion of growth factors, cytokines, and exosomes (Fig. 4c). The most relevant immunomodulatory function of UC-MSCs is considered to be their inhibition of effector T cells via the induction of T-cell apoptosis and cell cycle arrest by the production of indoleamine 2,3- dioxygenase (IDO), prostaglandin E2 (PGE-2), and TGF-β. Elevated levels of PGE-2 in patients with COVID-19 are reported to be a crucial factor in the initiation of inflammatory regulation by UC-MSCs post administration and prevent the development of cytokine storms by direct inhibition of T- and B lymphocytes.^403^ UC-MSCs exert these inhibitory activities through a PGE-2-dependent mechanism.^404^ It was reported that UC-MSCs confer the ability to secrete tolerogenic mediators, including TGF-β1, PGE-2, nitric oxide (NO), and TNF-α, which are directly involved in their immunoregulatory mechanism. The secretion of NO from UC-MSCs is reported to be associated with the desensitization of T cells via the IFN-inducible nitric oxide synthase (iNOS) pathways and to stimulate the migration of T cells in close proximity to MSCs that subsequently suppress T-cell sensitivities via NO.^405^ Lung infection with viruses usually leads to impairments in alveolar fluid clearance and protein permeability. The administration of UC-MSCs enhances alveolar protection and restores fluid clearance in patients with COVID-19. UC-MSCs secrete growth factors associated with angiogenesis and the regeneration of pulmonary blood vessels and micronetworks, including angiotensin-1, VEGF, and HGF, which also reduce oxidative stress and prevent fibrosis formation in the lungs. These trophic factors have been identified as key players in the modulation of the microenvironment and promote pulmonary repair. Additionally, UC-MSCs are more effective than BM-MSCs in the restoration of impaired alveolar fluid clearance and the permeability of airways in vitro, supporting the use of UC-MSCs in the treatment of patients with pulmonary pneumonia.^406^ In the context of pulmonary regeneration, UC-MSCs were shown to inhibit apoptosis and fibrosis in pulmonary tissue by activating the PI3K/AKT/mTOR pathways via the secretion of HGF, which also acts as an inhibitory stimulus that blocks alveolar epithelial-to-mesenchymal transition.^407,408^ Moreover, UC-MSCs can reverse the process of fibrosis via enhanced expression of macrophage matrix-metallopeptidase-9 for collagen degradation and facilitate alveolar regeneration via Toll-like receptor-4 signaling pathways.^409^ UC-MSCs were shown to communicate with CD4+ T cells through HGF induction not only to inhibit their differentiation into Th17 cells, reducing the secretion of IL-17 and IL-22 but also to switch their differentiation into regulatory T cells.^410,411^ In addition, UC-MSCs conferred the ability to facilitate the number of M2 macrophages and reduce M1 cells via the control of the macrophage polarization process.^412^

There are several potential mechanisms of UC-MSCs in the treatment of patients with pulmonary diseases and pneumonia, including the regulation of immune cell function, immunomodulation, the enhancement of alveolar fluid clearance and protein permeability, the modulation of endoplasmic reticulum stress, and the attenuation of pulmonary fibrosis. Hence, based on these discussions, UC-MSCs are recommended as suitable candidates for the treatment of pulmonary disease both in pediatric and adult patients.

## Proposed mechanism of AT-MSCs in the treatment of endocrinological diseases, reproductive disorders, and skin burns

**Fig. 5:**
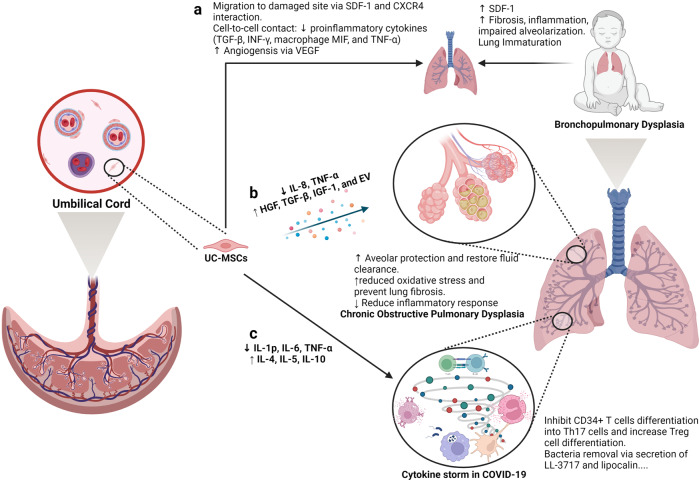
Umbilical cord-derived mesenchymal stem cells (UC-MSCs) are good candidates for the treatment of pulmonary diseases. **a** Lung immaturity and fibrosis are the major problems of patients with bronchopulmonary dysplasia and lead to increased levels of SDF-1, the development of fibrosis, the induction of the inflammatory response, and the impairment of alveolarization. UC-MSCs are attracted to the damaged lung via the chemoattractant SDF-1, which is constantly released from the immature lung via SDF-1 and CXCR4 communication. Moreover, UC-MSCs reduce the level of proinflammatory cytokines (TGF-β, INF-γ, macrophage MIF, and TNF-α) via a cell-to-cell contact mechanism. The ability of UC-MSCs to produce and secrete VEGF also involves in the regeneration of the immature lung through enhanced angiogenesis. **b** Upon an exacerbation of chronic obstructive pulmonary disease (COPD), UC-MSCs respond to the surrounding stimuli by reducing IL-8 and TNF-α levels, resulting in the inhibition of the inflammatory response but an increase in the secretion of growth factors participating in the protection of alveoli, fluid clearance and reduced oxidative stress and lung fibrosis, including HGF, TGF-β, IGF-1, and exosomes. **c** In a similar manner, UC-MSCs prevent the formation of cytokine storms in coronavirus disease 2019 (COVID-19) by inhibiting CD34+ T-cell differentiation into Th17 cells and enhancing the number of regulatory T cells. Moreover, UC-MSCs also have antibacterial activity by secreting LL-3717 and lipocalin. Figure was created with BioRender.com

Human AT was first viewed as a passive reservoir for energy storage and later as a major site for sex hormone metabolism, the production of endocrine factors (such as adipsin and leptin), and a secretion source of bioactive peptides known as adipokines.^413^ It is now clear that AT functions as a complex and highly active metabolic and endocrine organ, orchestrating numerous different biological features^414^ (Fig. 5a). In addition to adipocytes, AT contains hematopoietic-derived progenitor cells, connective tissue, nerve tissue, stromal cells, endothelial cells, MSCs, and pericytes. AT-MSCs and pericytes mobilize from their perivascular locations to aid in healing and tissue regeneration throughout the body. As AT is involved directly in energy storage and metabolism, AT-MSCs are also mediated and regulated by growth factors related to these pathways. In particular, interleukin-6 (IL-6), IL-33, and leptin regulate the maintenance of metabolic activities by increasing insulin sensitivity and preserving homeostasis related to AT. Nevertheless, in the development of obesity and diabetes, omental and subcutaneous AT maintains a low-grade state of inflammation, resulting in the impairment of glucose metabolism and potentially contributing to the development of insulin resistance.^415^ In normal AT, direct regulation of Pre-B-cell leukemia homeobox (Pbx)-regulating protein-1 (PREP1) by leptin and thyroid growth factor-beta 1 (TGF-β1) in AT-MSCs and mature adipocytes is involved in the protective function and maintenance of AT homeostasis. However, under diabetic conditions, the balance between the expression of leptin and the secretion of TGF-β1 is compromised, resulting in the malfunction of AT-MSC metabolic activity and the proliferation, differentiation, and maturation of adipocytes. Therefore, the use of autologous AT-MSCs in the treatment of diabetic conditions is not a suitable option, as the functions of AT-MSCs are directly altered by diabetic conditions, which reduces their effectiveness in cell-based therapy (Fig. 5b).

Preclinical studies and clinical trials have revealed the therapeutic effects of MSCs, in general, and AT-MSCs, in particular, in the management of POF, with relatively high efficacy and enhanced regeneration of the ovaries. Understanding the molecular and cellular mechanisms underlying these effects is the first step in the development of suitable MSC-based therapies for POF. One of the mechanisms by which MSCs exert their therapeutic effects is their ability to migrate to sites of injury, a process known as “homing”. Studies have shown that MSCs from different sources have the ability to migrate to different compartments of the injured ovary. For example, BM-MSCs administered through IV routes migrated mostly to the ovarian hilum and medulla,^416^ whereas a significant number of UC-MSCs were found in the medulla.^417^ Interestingly, AT-MSCs were found to be engrafted in the theca layers of the ovary but not in the follicles, where they acted as supportive cells to promote follicular growth and the regeneration of thecal layers.^418^ The structure and function of the thecal layer have a great impact on fertility, which has been reviewed elsewhere.^419^ In brief, the thecal layer consists of two distinct parts, the theca interna, which contains endocrine cells, and the theca externa, which is an outer fibrous layer. The thecal layer contains not only endocrine-derived cells but also vascular- and immune-derived cells, whose functions are to maintain the structural integrity of the follicles, transport nutrients to the inner compartment of the ovary and produce key reproductive hormones such as androgens (testosterone and dihydrotestosterone) and growth factors (morphogenic proteins, e.g., BMPs and TGF-β).^420^ As AT-MSCs originate from an endocrine organ, their ability to sense signals and migrate to the thecal layer is anticipated. Additionally, secretome analysis of AT-MSCs showed a wide range of growth factors, including HGF, TBG-β, VEGF, insulin-like growth factor-1 (IGF-1), and EGF,^421^ that are directly involved in the restoration of the structure and function of damaged ovaries by stimulating cell proliferation and reducing the aging process of oocytes via the activation of the SIRT1/FOXO1 pathway, a key regulator of vascular endothelial homeostasis.^422,423^ In POF pathology, autophagy and its correlated oxidative stress contribute to the development of POF throughout a patient’s life. Recently, AT-MSCs were shown to be able to improve the structure and function of mouse ovaries by reducing oxidative stress and inflammation, providing essential data supporting the mechanism of AT-MSCs in the treatment of POF.^424^ Several studies have illustrated that AT-MSCs secrete biologically active EVs that regulate the proliferation of ovarian granulosa cells via the PI3K/AKT pathway, resulting in the enhancement of ovarian function.^425^ Direct regulation of ovarian cell proliferation modulates the state of these cells, which in turn restores the ovarian reserve.^426^ Other mechanisms supporting the effectiveness of MSCs have been carefully reviewed, confirming the therapeutic potential of MSCs derived from different sources^426^ (Fig. 5c).

In the last decade, the number of clinical trials using AT-MSCs in the treatment of chronic skin wounds and skin regeneration has exponentially increased, with data supporting the enhancement of the skin healing processes, the reduction of scar formation, and improvements in skin structure and quality. Several mechanisms are directly linked to the origin of AT-MSCs, including differentiation ability, neovascularization, anti-apoptosis, and immunological regulation. AT is a connective and supportive tissue positioned just beneath the skin layers. AT-MSCs have a strong ability to differentiate into adipocytes, endothelial cells,^427^ epithelial cells^428^ and muscle cells.^429^ The adipogenic differentiation of AT-MSCs is one of the three mesoderm lineages that defines MSC features, and AT-MSCs are likely to be the best MSC type harboring this ability compared to BM- and UC-MSCs. Recent reports detailed that AT-MSCs accelerated diabetic wound tissue closure through the recruitment and differentiation of endothelial cell progenitor cells into endothelial cells mediated by the VEGF-PLCγ-ERK1/ERK2 pathway.^430^ Upon injury, the skin must be healed as quickly as possible to prevent inflammation and excessive blood loss. The reparation process occurs through distinct overlapping phases and involves various cell types and processes, including endothelial cells, keratinocyte proliferation, stem cell differentiation, and the restoration of skin homeostasis.^431^ Hence, the differentiation ability of AT-MSCs plays a critical role in their therapeutic effect on skin wound regeneration and healing processes. AT-MSCs accelerate wound healing via the production of exosomes that serve as paracrine factors. It was reported that AT-MSCs responded to skin wound injury stimuli by increasing their expression of the lncRNA H19 exosome, which upregulated SOX9 expression via miR-19b, resulting in the acceleration of human skin fibroblast proliferation, migration, and invasion.^432^ In addition, the engraftment of AT-MSCs supported wound bed blood flow and epithelialization processes.^433^ Anti-apoptosis plays a critical role in AT-MSC-based therapy, as without a microvascular supply network established within 4 days post injury, adipocytes undergo apoptosis and degenerate. Exogenous sources of AT-MSCs mediate anti-apoptosis via IGF-1 and exosome secretion by triggering the activation of PI3K signaling pathways.^434^ Another mechanism supporting the therapeutic potential of AT-MSCs is their anti-inflammatory function, which results in the reduction of proinflammatory factors, such as tumor necrosis factor (TNF) and interferon-γ (IFN-γ), and increases the production of the anti-inflammatory factors IL-10 and IL-4. Exosomes from AT-MSCs in response to a wound environment were found to contain high levels of Nrf2, which downregulated wound NADPH oxidase isoform 1 (NOX1), NADPH oxidase isoform 4 (NOX4), IL-1β, IL-6, and TNF-α expression. The anti-inflammatory functions of AT-MSCs are also regulated by their immunomodulatory ability, partially through the inhibition of NF-κB activation in T cells via the PD-L1/PD-1 and Gal-9/TIM-3 pathways, providing a novel target for the acceleration of wound healing^435^ (Fig. 5d).

Therefore, as an endocrine organ in the human body, AT and its derivative stem cells, including AT-MSCs, have shown great potential in the treatment of reproductive disorders and skin diseases. Their potential is supported by mechanisms that are directly related to the nature of AT-MSCs in the maintenance of tissue homeostasis, angiogenesis, anti-apoptosis, and the regulation of inflammatory responses.

## The current challenges for MSC-based therapies

Over the past decades, MSC-based research and therapy have made tremendous advancements due to their advantages, including immune evasion, diverse tissue sources for harvesting, ease of isolation, rapid expansion, and cryopreservation as “off-the-shelf” products. However, several important challenges have to be addressed to further enhance the safety profile and efficacy of MSC-based therapy. In our opinion, the most important challenge of MSC-based therapy is the fate of these cells post administration, especially the long-term survival of allogeneic cells in the treatment of certain diseases. Although reported data confirm that the majority of MSCs are trapped in the lung and rapidly removed from the circulation, caution has been raised related to the occurrence of embolism events post infusion, which was proven to be related to MSC-induced innate immune attack (called instant blood-mediated inflammatory reaction).^436^ Another related challenge is the homing ability of infused cells, as successful homing at targeted tissue might result in long-term benefits to patients. Other concerns related to MSC-based therapy are the number of dead cells infused into the patients. An interesting study reported that dead MSCs alone still exerted the same immunomodulatory property as live MSCs by releasing phosphatidylserine.^437^ This is an interesting observation, as there is always a certain number of dead cells present in the cell-based product, and concerns are always raised related to their effects on the patient’s health. Finally, the hypothesis presented in this review is also a great challenge of the field, which has been proposed for future studies to answer the question: “What is the impact of MSC sources on their downstream application?”. Tables 5 and 6 illustrate the comparative studies that were conducted in preclinical and clinical settings to address the MSC source challenge. Other challenges of MSC-based therapies have been discussed in several reviews and systematic studies,^135,185,438,439^ which are highly recommended.

**Table 5:** Comparative analysis of the effectiveness of MSC sources in a preclinical setting

Study name	Time	Disease	Intervention			Treatment outcomes	Effective cell sources
			Source	Dose	Route		
Comparison of Transplantation of Bone Marrow Stromal Cells (BMSC) and Stem Cell Mobilization by Granulocyte Colony-Stimulating Factor after Traumatic Brain Injury in Rat.^490^	2010	Traumatic Brain Injury	BM-MSCs and bone marrow hematopoietic stem cell mobilization, induced by granulocyte colony-stimulating factor (G-CSF)	2 × 10^6^ cells	IV	Significant improvement in functional outcome. The recovery of motor and sensory score was faster and more tangible after injection of BMSC and G-CSF.	No significant difference between two sources.
Comparison of mesenchymal stromal cells from human bone marrow and adipose tissue for the treatment of spinal cord injury^491^	2013	Spinal cord injury	Human BM-MSCs vs. AT-MSCs	2 × 10^5^ cells	Inject into injured spinal cord	Increase BDNF levels in the injured spinal cord, reduce lesion cavity volume and microglia/macrophage infiltration Induce angiogenesis, axonal regeneration Improve behavioral performance.	Both AT- and BM-MSCs migrated to the injured spinal cord without differentiating into glial or neuronal cells. AT-MSCs significantly increases BDNF levels more than in BM-MSCs group.
Human bone marrow-derived and umbilical cord-derived mesenchymal stem cells for alleviating neuropathic pain in a spinal cord injury model.^492^	2016	Spinal cord injury	Human BM-MSCs vs. UC-MSCs	1 × 10^6^ cells	Dorsal horn of the spinal cord injection	MSC administration resulted in improving functional recovery, allodynia, and hyperalgesia.	BM-MSCs and UC-MSCs show similar effectiveness in alleviating the symptoms of neuropathic pain and improve motor function. Survival rate and electrophysiological findings of UC-MSC were significantly better than BM-MSC.
Comparison of Mesenchymal Stromal Cells Isolated from Murine Adipose Tissue and Bone Marrow in the Treatment of Spinal Cord Injury.^493^	2018	Spinal cord injury	Mice AT-MSCs vs. BM-MSCs	1 × 10^5^ cells	T9-T10 total laminectomy	Promoted axonal regeneration and blood vessel formation in injury epicenter. Improved the motor function.	Survival rate of AT-MSCs was higher than BM-MSCs. AT-MSCs transplantation protected neuronal/vascular better than BM-MSCs The motor function was equally improved following moderate spinal cord injury in both groups. No significant improvement in the severe spinal cord in either group.
Comparison of bone marrow-vs. adipose tissue-derived mesenchymal stem cells for attenuating liver fibrosis.^494^	2017	Liver fibrosis	Rat AT-MSCs vs. BM-MSCs	5 × 10^6^ cells	portal vein	Attenuate liver fibrosis by inhibiting the activation and proliferation of Hepatic stellate cells (HSCs), as well as promoting the apoptosis of HSCs.	No significant difference between two sources.
Bone marrow or adipose tissue mesenchymal stem cells: Comparison of the therapeutic potentials in mice model of acute liver failure.^495^	2018	Acute liver failure	Murine AT-MSCs vs. BM-MSCs.	1 × 10^6^ cells	IV	Improved histopathological score of liver tissue, reduced serum concentration of ALT and AST, and led to increase in survival rate.	AT-MSCs were more effective in preventing hepatic enzyme rise (lower level of aspartate aminotransferase (AST) and alanine aminotransferase (ALT)).
Mesenchymal stem cells provide better results than hematopoietic precursors for the treatment of myocardial infarction.^496^	2010	Myocardial infarction	Human BM-MSCs vs. Human CD34+ Hematopoietic stem cells.	1.2 × 10^6^ BM-MSCs 6 × 10^5^ CD34+ cells	IV	Both cell types induced an improvement in LV cardiac function and increased tissue cell proliferation in myocardial tissue and neoangiogenesis. However, MSC were more effective for the reduction of infarct size and prevention of ventricular remodeling.	Mesenchymal stem cells might be more effective than CD34+cells for the healing of the infarct
Cell origin of human mesenchymal stem cells determines a different healing performance in cardiac regeneration^497^	2011	Myocardial infarction	AT-MSCs BM-MSCs UC-MSCs	4 × 10^5^ cells/animal	intramyocardial injection	The findings suggests that hMSC originating from different sources showed a different healing performance in cardiac regeneration.	CD105+ hMSC exhibited a favorable survival pattern in infarcted hearts, which translates into a more robust preservation of cardiac function
Mesenchymal stromal cells mediate a switch to alternatively activated monocytes/macrophages after acute myocardial infarction^498^	2011	Myocardial infarction	human umbilical cord perivascular cells vs. hBM-MSCs	2 × 10^6^ cells	IV	In the presence of either cell types, overall macrophage/monocyte levels were reduced, including proinflammatory M1-type macrophages, while the proportion of alternatively activated M2-type macrophages was significantly increased in the circulation and heart but not the BM. Moreover, we found decreased expression of IL-1β and IL-6, increased IL-10 expression, and fewer apoptotic cardiomyocytes without changes in angiogenesis in the infarct area. Fractional shortening was enhanced 2 weeks after cell infusion but was similar to medium controls 16 weeks after MI.	No significant difference between two sources.
Wharton’s jelly or bone marrow mesenchymal stromal cells improve cardiac function following myocardial infarction for more than 32 weeks in a rat model: a preliminary report^499^	2013	Myocardial infarction	Mouse Wharton’s jelly MSCs vs. Mouse BM-MSCs.	4–10 × 10^6^ cells	IV	MSCs administered at 24–48 hr after MI have a significant and durable beneficial effect more than 25 weeks after MI and that MSC treatment can home to damaged tissue and improve heart function after intravenous infusion 24–48 hrs after MI, and that WJCs may be a useful source for off-the-shelf cellular therapy for MI.	WJCs might be more advantageous than BM-MSCs as an allogeneic cell source.
Comparison of human adipose-derived stem cells and bone marrow-derived stem cells in a myocardial infarction model.^500^	2014	Myocardial infarction	AT-MSCs BM-MSCs	1 × 10^6^ cells	intramyocardial injection	After 4 weeks, left ventricular ejection fraction (LVEF) was improved in the adipose-derived stem cell group, and scar wall thickness was greater compared with the saline group. Adipose-derived, as well as bone marrow-derived mesenchymal stem cells, prevented left ventricular end-diastolic dilation	Adipose-derived stem cells from a human ischemic patient preserved cardiac function following MI, whereas this could not be demonstrated for bone marrow-derived mesenchymal stem cells, with only adipose-derived stem cells leading to an improvement in LVEF.
Mechanical and Chemical Predifferentiation of Mesenchymal Stem Cells Into Cardiomyocytes and Their Effectiveness on Acute Myocardial Infarction^501^	2018	Myocardial infarction	AT-MSCs BM-MSCs	1 × 10^6^ cells	intramyocardial injection	The results demonstrated significant scar size reduction and greater recovery of left ventricle ejection fraction after transplantation of predifferentiated cells, although the differences were not significant when comparing mechanically with chemically predifferentiated MSCs.	No significant difference between two sources.
Comparative Study of the Therapeutic Potential of Mesenchymal Stem Cells Derived from Adipose Tissue and Bone Marrow on Acute Myocardial Infarction Model^502^	2019	Myocardial infarction	AT-MSCs BM-MSCs	2 × 10^6^ cells	intramyocardial injection	MSC therapy repaired cardiac functions shown by the restoration of ST segment, QT and QRS intervals in the ECG when compared to the AMI group. Infarct area was significantly decreased, and cardiac tissue regeneration signs were shown on histopathological examination.	No significant difference between two sources.

**Table 6:** Clinical trials comparing the efficacy of MSCs derived from different sources in the treatment of pulmonary diseases and cardiovascular conditions

Study name	Time	Disease	Intervention			Treatment outcomes	Effective cell sources
			Source	Dose	Route		
Transendocardial mesenchymal stem cells and mononuclear bone marrow cells for ischemic cardiomyopathy: the TAC-HFT randomized trial^315^ (NCT00768066)	2014	Ischemic Heart Failure	BM-MSCs Vs. Autologous bone marrow mononuclear cells (aBMNCs)	2 × 10^8^ cells	Transendocardial (TE) injection	TE injection of BM-MSCs or aBMNCs appeared to be safe for patients with chronic cardiomyopathy and left ventricular dysfunction.	No significant difference between two approaches.
Randomized Comparison of Allogeneic Versus Autologous Mesenchymal Stem Cells for Nonischemic Dilated Cardiomyopathy: POSEIDON-DCM Trial^310^ (NCT01392625)	2017	Nonischemic Dilated Cardiomyopathy (NIDCM)	Autologous BM-MSCs vs. Allogeneic BM-MSCs.	1 × 10^8^ cells	Transendocardial (TE) injection	The results support the safety profiles of BM-MSCs in the treatment of NIDCM patients.	Allogeneic BM-MSCs
Intramyocardial Transplantation of Umbilical Cord Mesenchymal Stromal Cells in Chronic Ischemic Cardiomyopathy: A Controlled, Randomized Clinical Trial (HUC-HEART Trial)^325^ (NCT02323477)	2020	Myocardial Infarction	Allogeneic UC-MSCs vs. bone marrow mononuclear cells (aBMNCs)	2 × 10^7^ UC-MSCs 20–25 × 10^7^ aBMNCs	intramyocardial injection	Significant results were observed in the intramyocardial delivery of UC-MSCs justified their efficacy in chronic ischemic cardiomyopathy.	UC-MSCs
Autologous Infusion of Bone Marrow and Mesenchymal Stromal Cells in Patients with Chronic Obstructive Pulmonary Disease: Phase I Randomized Clinical Trial^315^ (NCT02412332)	2021	Chronic Obstructive Pulmonary Disease	Autologous bone marrow mononuclear cells (aBMNCs) vs. Coinfusion of BMNCs and AT-MSCs.	1 × 10^8^ cells	IV	Safety: No adverse events Efficiency: - BMMC group showed an increase in forced expiratory volume (FEV1) and diffusing capacity for carbon monoxide (DLCO). - Coinfusion group showed a DLCO, and gas exchange improvement and a better quality of life.	No significant difference between two approaches.

## Limitations of the current hypothesis

The proposed hypothesis presented in this review was made based on (1) the calculated number of recovered patients from published clinical trials; (2) the empirical experience of the authors in the treatment of brain-related diseases,^440^ pulmonary disorders,^215^ and endocrinological conditions;^271,441^ and (3) the proposed mechanisms by which each type of MSC exhibits its best potential for downstream applications. The authors understand that the approach that we used has a certain level of research bias, as a comprehensive meta-analysis is needed to first confirm the correlation between the origins of MSCs and their downstream clinical outcomes before a complete hypothesis can be made. However, to date, a limited number of clinical trials have been conducted to directly compare the efficacy of MSCs from different sources in treating the same disease, which in turn dampened our analysis to prove this hypothesis. In addition, MSC-based therapy is still in its early stages, as controversy and arguments are still present in the field, including (1) the name of MSCs (medicinal signaling cells vs. MSCs or mesenchymal stromal cells),^442,443^ (2) the existence of “magic cells” (one cell type for the treatment of all diseases),^444,445^ (3) the conflicting results from large-scale clinical trials,^135^ and (4) the dangerous issues of unauthorized, unproven stem cell therapies and clinics.^446,447^ Therefore, our hypothesis is proposed at this time to encourage active researchers and clinicians to either prove or disprove it so that future research can strengthen the uses of MSC-based therapies with solid mechanistic study results and clarify results for “one cell type for the treatment of all diseases”.

Another limitation is the knowledge coverage in the field of MSC-based regenerative medicine, as discussed in this study. First, the abovementioned diseases were narrowed to four major disease categories for which MSC-based therapy is widely applied, including neuronal, pulmonary, cardiovascular, and endocrinological conditions. In fact, other diseases also receive great benefits from MSC therapy, including liver cirrhosis,^448^ bone regeneration,^360^ plastic surgery,^449^ autoimmune disease,^450^ etc., which are not fully discussed in this review and included in our hypothesis. Recently, the secretome profile of MSCs and its potential application in clinical settings have emerged as a new player in the field, with a recently published comprehensive review including MSC-derived exosomes.^451,452^ To date, the therapeutic potential of MSCs is believed to be strongly influenced by their secretomes, including growth factors, cytokines, chemokines, and exosomes.^453^ However, this body of knowledge is also not fully included in our discussion, as this review focuses on the function and potency of MSCs as a whole with considerations derived from published clinical data. Therefore, the authors believe in and support the future applications of the secreted components derived from MSCs, including exosomes, in the treatment of human diseases. In fact, this potential approach could elevate the uses of MSCs to the next level, where the sources of MSCs could be neglected with advancements in the development of protocols that allow strict control of the secretome profiles of MSCs under specific conditions.^454–456^ Finally, strategies that could potentially enhance the therapeutic outcomes of MSC-based therapy, such as the “priming” process, are not discussed in this review. The idea of “priming” MSCs is based on the nature of MSCs, which is similar to the immune cells,^457^ that MSCs have proven to be able to “remember” the stimulus from the surrounding environment.^458,459^ Thus, activating or priming MSCs using certain conditions, such as hypoxia, matrix mechanics, 3D environment, hormones, or inflammatory cytokines, could trigger the memory mechanism of the MSCs in vitro so that these cells are ready to function towards specific therapeutic activities without the need for in vivo activation.^3,460^

## Conclusion

From a cellular and molecular perspective and from our own experience in a clinical trial setting, AD-, BM- and UC-MSCs exhibit different functional activities and treatment effectiveness across a wide range of human diseases. In this paper, we have provided up-to-date data from the most recently published clinical trials conducted in neuronal diseases, endocrine and reproductive disorders, skin regeneration, pulmonary dysplasia, and cardiovascular diseases. The implications of the results and discussions presented in this review and in a very large body of comprehensive and excellent reviews as well as systematic analyses in the literature provide a different aspect and perspective on the use of MSCs from different sources in the treatment of human diseases. We strongly believe that the field of regenerative medicine and MSC-based therapy will benefit from active discussion, which in turn will significantly advance our knowledge of MSCs. Based on the proposed mechanisms presented in this review, we suggest several key mechanistic issues and questions that need to be addressed in the future:

1. The confirmation and demonstration of the mechanism of action prove that tissue origin plays a significant role in the downstream applications of the originated MSCs.
2. Is it required that MSCs derived from particular cell sources need to have certain functionalities that are unique to or superior in the original tissue sources?
3. As mechanisms may rely on the secretion of factors from MSCs, it is important to identify the specific stimuli from the wound environments to understand how MSCs from different sources can exhibit similar functions in the same disease and whether or not MSCs derived from a particular source have stronger effects than their counterparts derived from other tissue sources.
4. Should we create “universal” MSCs that could be functionally equal in the treatment of all diseases regardless of their origin by modeling their genetic materials?
5. Can new sources of MSCs from either perinatal or adult tissues better stimulate the innate mechanisms of specific cell types in our body, providing a better tool for MSC-based treatment?
6. A potential ‘priming’ protocol that allows priming, activating, and switching the potency of MSCs from one source to another with a more appropriate clinical phenotype to treat certain diseases. This idea is potentially relevant to our suggestion that each MSC type could be more beneficial in downstream applications, and the development of such a “priming” protocol would allow us to expand the bioavailability of specific MSC types.

**Fig. 6:**
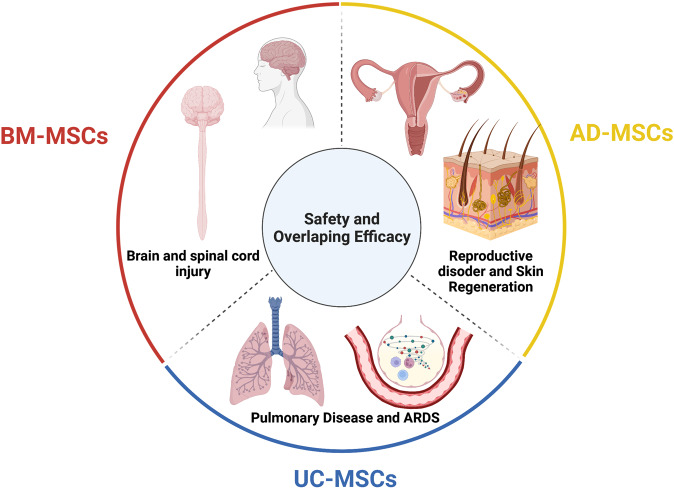
The tissue sources of mesenchymal stem cells (MSCs) contribute greatly to their therapeutic potential, as all MSC types share safety profiles and overlapping efficacy. Although a large body of data and their review and systematic analysis indicated the shared safety and potential efficacy of MSCs derived from different tissue sources, targeted therapies considering MSC origin as an important factor are imperative to enhance the downstream therapeutic effects of MSCs. We suggest that bone marrow-derived MSCs (BM-MSCs) are good candidates for the treatment of brain and spinal cord injury, adipose tissue-derived MSCs (AT-MSCs) are suitable for the treatment of reproductive disorders and skin regeneration, and umbilical cord-derived MSCs (UC-MSCs) could be alternatives for the treatment of pulmonary diseases and acute respiratory distress syndrome (ARDS). Figure was created with BioRender.com

From our clinical perspective, the underlying proposal in our review is to no longer use MSCs for applications while disregarding their sources but rather to match the MSC tissue source to the application, shifting from one cell type for the treatment of all diseases to cell source-specific disease treatments. Whether the application of MSCs from different sources still shows their effectiveness to a certain extent in the treatment of diseases or not, the transplantation of MSCs derived from different sources for each particular disease needs to be further investigated, and protocols need to be established via multicentre, randomized, placebo-controlled phase II and III clinical trials (Fig. 6).

## Data Availability

All data generated or analyzed in this study are included in this published article.
